# Biocompatible Stimuli-Sensitive Natural Hydrogels: Recent Advances in Biomedical Applications

**DOI:** 10.3390/gels11120993

**Published:** 2025-12-10

**Authors:** Jose M. Calderon Moreno, Mariana Chelu, Monica Popa

**Affiliations:** “Ilie Murgulescu” Institute of Physical Chemistry, 202 Spl. Independentei, 060021 Bucharest, Romania; calderon@icf.ro

**Keywords:** natural biocompatible polymers, multi-stimuli responsive biomaterials, smart and dynamic hydrogels, sustainable healthcare, health monitoring biosensors, wound healing and tissue engineering, controlled drug delivery, chitosan and alginate composites, injectable and in situ forming gels

## Abstract

Biocompatible stimuli-sensitive hydrogels are a versatile and promising class of materials with significant potential for various biomedical applications. These ‘’smart’’ hydrogels can dynamically respond to external environmental stimuli such as pH, temperature, enzymes, or biomolecular interactions, enabling controlled drug release, tissue regeneration, wound healing, and biosensing applications. Hydrogels derived from natural polymers, including chitosan, alginate, collagen, and hyaluronic acid, offer key advantages such as intrinsic biocompatibility, biodegradability, and the ability to mimic the extracellular matrix. Their ability to respond to environmental stimuli—including pH, temperature, redox potential, and enzymatic activity—enables control over drug release and tissue regeneration processes. This review explores the fundamental principles governing the design, properties, and mechanisms of responsiveness of natural stimuli-sensitive hydrogels. It also highlights recent advancements in their biomedical applications, discusses existing challenges, and outlines future research directions aimed at improving their functional performance and therapeutic potential for sustainable healthcare solutions.

## 1. Introduction

**Figure 1:**
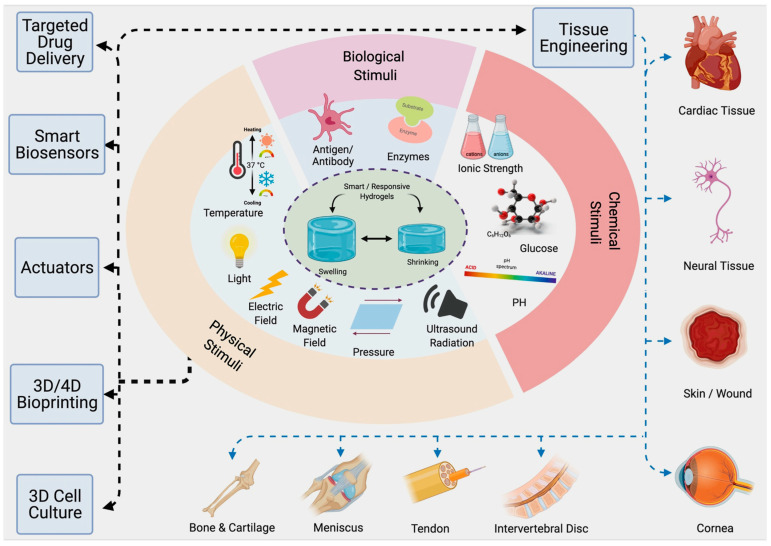
Schematic illustration of smart/stimuli-responsive hydrogels employed for different biomedical applications. Reproduced from [9].

Hydrogels are three-dimensional polymeric networks capable of retaining large amounts of water while maintaining structural integrity. Their high-water content, soft texture and tunable properties enable close mimicry of natural tissues, making them key materials in biomedical applications [1], such as drug delivery, tissue engineering and wound healing [2,3,4,5,6,7,8]. Among various hydrogel systems, stimuli-sensitive or “smart” hydrogels have attracted growing attention for their ability to undergo reversible physical or chemical changes in response to environmental cues, including pH, temperature, redox potential, enzymatic activity and biomolecular interactions (Figure 1) [9]. This responsiveness allows for on-demand control over swelling, permeability and drug release, supporting the development of adaptive and personalized therapeutic systems [10].

Natural polymer-based hydrogels—derived from chitosan, alginate, collagen, gelatin, hyaluronic acid (HA), cellulose, dextran and silk fibroin—offer distinct advantages over synthetic analogues. Their inherent biocompatibility, biodegradability and structural similarity to the extracellular matrix (ECM) promote cellular adhesion, proliferation and differentiation [11].

Moreover, the presence of reactive functional groups in natural polymers facilitates chemical modification and cross-linking, allowing fine-tuning of responsiveness and mechanical strength [12]. Because they originate from renewable resources and degrade into nontoxic by-products, natural hydrogels are particularly suitable for clinical and translational applications [13]. Stimuli-responsive natural hydrogels merge the biocompatibility of natural polymers with dynamic adaptability. For example, pH-sensitive chitosan and alginate systems respond to local acidity variations, making them valuable for tumour-targeted or oral drug delivery [6,14]. Temperature-sensitive gelatin and collagen hydrogels undergo sol–gel transitions near physiological temperatures, supporting injectable and in situ forming scaffolds for minimally invasive tissue repair [15,16,17]. Enzyme- and redox-responsive systems can release therapeutics in response to disease-related biochemical signals, while multi-stimuli-responsive designs integrate several mechanisms for greater control and selectivity [18,19,20].

These materials are finding widespread use in controlled drug release, tissue regeneration, wound healing and biosensing. Their hydrated structure and tunable porosity facilitate nutrient transport and cell–matrix interactions, whereas stimuli responsiveness allows synchronization with physiological environments—such as responding to the acidic tumour microenvironment, elevated reactive oxygen species (ROS) levels in wounds, or temperature changes in implanted systems [21]. Functionalizing natural hydrogels to impart advanced properties, such as responsiveness to pH, temperature, or biological stimuli, is crucial for expanding their applications in fields like drug delivery, tissue engineering, and biosensing. However, achieving this functionalization without compromising the hydrogel’s structural integrity poses a major technical challenge [22]. Natural hydrogels often suffer from limited mechanical strength, variability in composition, and scale-up challenges. Balancing biocompatibility with durability and reproducibility remains a key obstacle to clinical translation [23].

Recent progress in hybrid and nanocomposite hydrogels, self-healing systems and 3D-bioprinted scaffolds has begun to address these limitations. Integration of computational modeling (CM) and machine learning (ML) tools is further enabling the rational design of hydrogels with predictive performance under physiological conditions [24,25]. These advances are pushing the field toward next-generation smart biomaterials capable of real-time adaptation within biological systems.

Given the rapid progress in this field, a comprehensive understanding of the design principles, properties and biomedical relevance of biocompatible stimuli-sensitive natural hydrogels is essential. This review aims to present the fundamental mechanisms governing hydrogel responsiveness, structural and compositional aspects of natural polymers, and recent advancements in their wide-ranging biomedical applications. Additionally, current limitations and future perspectives are discussed to highlight opportunities for innovation and translation into clinically viable materials.

## 2. Fundamental Concepts of Natural Stimuli-Sensitive Hydrogels

### 2.1. Structure and Composition of Natural Polymers Used in Stimuli-Sensitive Hydrogels

Stimuli-responsive hydrogels are crosslinked, water-swollen polymeric networks that undergo physicochemical changes when exposed to environmental stimuli. When derived from natural polymers, they combine biocompatibility and biodegradability with dynamic responsiveness, enabling biomedical functions that mimic native extracellular matrices (ECMs) [26]. The main ones (Table 1) are:

#### 2.1.1. Chitosan-Based Hydrogels

Chitosan, obtained by the deacetylation of chitin, is a cationic polysaccharide featuring amino and hydroxyl groups that can form physical or covalent crosslinks. Its pH-responsiveness arises from protonation of the amino groups, allowing swelling in acidic conditions and deswelling in neutral or basic media [27,28]. Thermosensitive chitosan systems are also achieved through blending with poly(ethylene glycol) or β-glycerophosphate to form injectable gels that solidify at body temperature [9]. Despite their notable bioactivity and inherent antimicrobial characteristics, chitosan hydrogels commonly display insufficient mechanical robustness and accelerated degradation, driving efforts to enhance them through nanoparticle or secondary-polymer-based composites [17,27,28].

#### 2.1.2. Alginate-Based Hydrogels

Alginate, an anionic polysaccharide composed of β-D-mannuronic and α-L-guluronic acids, forms hydrogels through ionic crosslinking with divalent cations such as Ca^2+^. Its mild gelation conditions are compatible with living cells, making it a key material in encapsulation and tissue scaffolds [11,29,30]. Stimuli sensitivity can be introduced by pH-responsive carboxyl groups or redox-sensitive functionalization. However, alginate lacks intrinsic cell-adhesive motifs and exhibits limited mechanical stability, leading to hybrid systems where gelatin or collagen improves cell affinity and resilience [31,32].

#### 2.1.3. Collagen and Gelatin-Based Hydrogels

Collagen, the most abundant structural protein in the ECM [33], and its denatured derivative gelatin, offer natural cell adhesion sites such as RGD sequences [34]. These hydrogels are inherently enzyme-responsive, as they are susceptible to collagenase and matrix metalloproteinase (MMP) degradation [35]. Temperature-responsive sol–gel transitions of gelatin are exploited for in situ forming scaffolds. Despite high bioactivity, pure collagen or gelatin hydrogels suffer from weak mechanical strength and rapid enzymatic breakdown; thus, crosslinking with genipin, glutaraldehyde, or methacrylation is often used to tune stiffness and degradation rate [36].

#### 2.1.4. HA-Based Hydrogels

HA, a linear glycosaminoglycan composed of alternating N-acetyl-glucosamine and glucuronic acid units, plays a crucial role in ECM hydration and cell signaling. HA hydrogels are biocompatible, non-immunogenic, and biodegradable via hyaluronidase enzymes. Functionalization with thiol or aldehyde groups allows incorporation of redox- or enzyme-sensitive linkages, enabling on-demand degradation and release in oxidative or inflammatory environments [37,38]. HA’s hydrophilicity and ECM-mimicking structure make it ideal for wound healing, cartilage repair, and injectable regenerative therapies [39].

#### 2.1.5. Other Natural Polymers (Cellulose, Dextran, Silk Fibroin, etc.)

**Figure 2:**
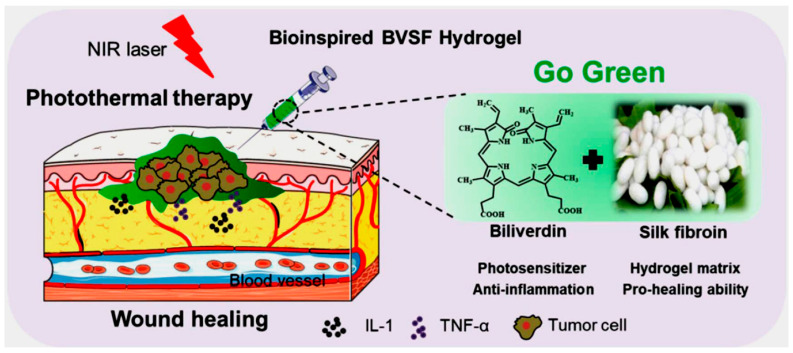
Design and fabrication of bioinspired biliverdin/silk fibroin hydrogel for photothermal antitumor therapy and wound healing. Reproduced from [42].

Other natural polymers, such as cellulose derivatives, dextran, and silk fibroin, broaden the functional landscape of biopolymer hydrogels. Cellulose nanocrystals reinforce hydrogel matrices, enhancing mechanical strength and shape stability [40,41]. A fully green hydrogel integrating biliverdin into a silk fibroin matrix developed for antiglioma photothermal therapy stimulated angiogenesis and wound healing in mice (Figure 2) [42]. Dextran-based hydrogels often incorporate enzyme- or redox-responsive linkers for controlled drug release, while silk fibroin provides exceptional toughness and tunable biodegradation through β-sheet crystallinity [43,44]. These materials can be combined with primary polymers (e.g., alginate or chitosan) to achieve synergistic responsiveness and biofunctionality [20].

### 2.2. Mechanisms of Stimuli Responsiveness

Stimuli-responsive hydrogels modulate their physicochemical behavior, such as swelling, porosity, crosslinking density, and sol–gel phase, upon exposure to specific external or internal triggers. These stimuli can be physical, chemical, and biological (Table 2) [9,40,45].

#### 2.2.1. pH-Responsive Systems

These systems contain ionizable moieties (-COOH, -NH_2_, -SO_3_H) whose charge states depend on environmental pH. Protonation or deprotonation alters electrostatic repulsion and osmotic pressure, leading to reversible volume changes. For example, chitosan swells under acidic conditions due to protonated amine groups, whereas alginate expands in alkaline media through carboxyl deprotonation [46]. This behavior enables site-specific delivery, such as oral or tumor-targeted therapeutics.

#### 2.2.2. Temperature-Responsive Systems

Thermosensitive hydrogels exhibit a lower or upper critical solution temperature (LCST/UCST), at which polymer-water interactions shift from hydrophilic to hydrophobic. Gelatin, methylcellulose, and chitosan/β-glycerophosphate systems exemplify natural or hybrid thermogels that transition near physiological temperatures (around 37 °C), facilitating minimally invasive injections [15,26]. Optimizing LCST and gelation kinetics is critical to avoid premature solidification or inadequate mechanical integrity in vivo.

#### 2.2.3. Enzyme-Responsive Systems

These hydrogels undergo degradation or sol–gel transition upon enzymatic cleavage of crosslinks or backbone chains. Collagen, gelatin, and HA naturally respond to MMPs, collagenase, or hyaluronidase, making them suitable for tissue remodeling and controlled drug release [31,47,48]. Synthetic peptide linkers are often introduced into natural polymer matrices to tailor responsiveness to disease-specific enzyme profiles.

#### 2.2.4. Redox-Responsive Systems

Redox-sensitive hydrogels exploit disulfide or thioketal linkages, which are cleaved under reductive intracellular environments rich in glutathione (GSH). Incorporating redox-labile bonds into HA, dextran, or silk fibroin networks allows precise release of drugs in oxidative stress-related diseases, including cancer and inflammation [49,50].

#### 2.2.5. Multi-Stimuli-Responsive Systems

Combining two or more responsive elements enhances specificity and control. For instance, a chitosan-gelatin composite may be both pH- and temperature-sensitive, while HA-based hybrids with disulfide and peptide crosslinks respond to redox and enzymatic cues simultaneously. Such multi-triggered systems are increasingly applied in precision medicine and biosensing, offering logical control over therapeutic outcomes [35,51].

**Figure 3:**
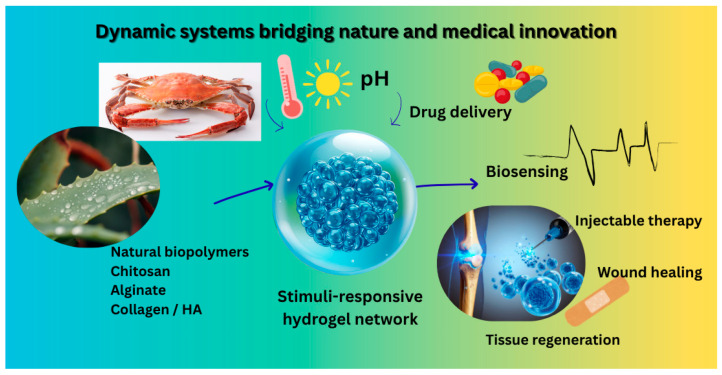
Schematic of natural polymer stimuli-responsive hydrogels and biomedical applications.

Figure 3 shows a central hydrogel matrix responding to multiple external stimuli: pH, temperature, redox, enzymatic, and biomolecular, leading to distinct applications: (i) controlled drug release (e.g., in tumours), (ii) in situ gelling for tissue regeneration, (iii) wound healing and antioxidant response, (iv) biosensing and diagnostics, and (v) injectable minimally invasive therapy. The figure highlights representative natural polymers (chitosan, alginate, collagen, HA, cellulose) and their functional behaviors within a physiological context.

### 2.3. Crosslinking Strategies: Physical vs. Chemical

**Figure 4:**
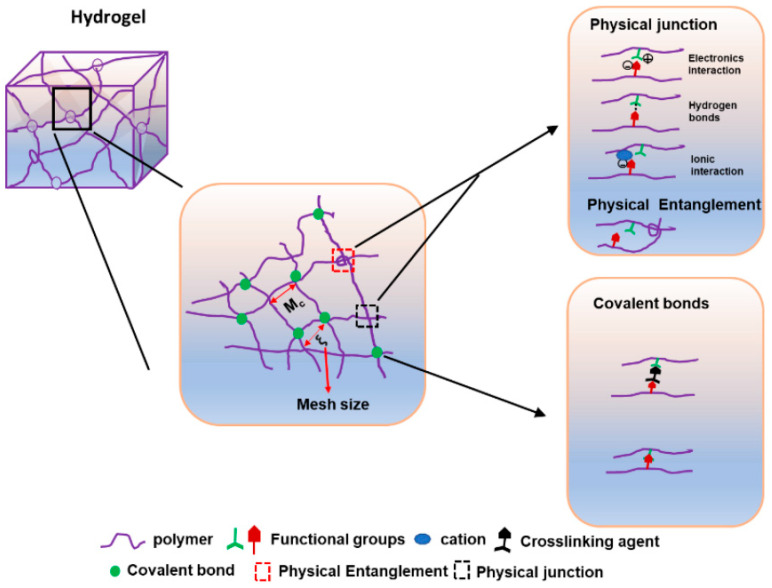
Crosslinking mechanisms in hydrogels. Reproduced from [1].

The crosslinking mechanism (Figure 4) determines hydrogel stability, reversibility, and responsiveness [1]. Physically crosslinked hydrogels rely on non-covalent interactions: hydrogen bonding, hydrophobic association, ionic interactions, or crystallization [11,52]. These systems are often reversible, cytocompatible, and easier to process, but may lack mechanical robustness. Alginate (Ca^2+^-mediated), gelatin (thermoreversible), and silk fibroin (β-sheet formation) are typical examples [53,54,55]. Chemically crosslinked hydrogels involve covalent bonds formed via enzymatic, photochemical, or click reactions [56,57]. While they yield stronger and more durable matrices, harsh reaction conditions or residual reagents can reduce biocompatibility. Advanced “dual crosslinking” strategies -combining reversible physical and permanent chemical networks- offer a balance between flexibility and mechanical strength [58]. Selecting a crosslinking strategy thus depends on the desired mechanical profile, degradation kinetics, and sensitivity to environmental triggers (Table 3).

### 2.4. Factors Influencing Hydrogel Behavior and Performance

The behavior and overall performance of stimuli-sensitive natural hydrogels arise from a complex interplay of compositional, structural, and environmental factors that collectively dictate their physicochemical response [59]. Polymer composition is a primary determinant, as the chemical functionality and molecular architecture of biopolymers govern chain interactions, water affinity, and the availability of moieties that can participate in reversible bonding or crosslinking reactions. For example, hydrogels built from polysaccharides such as alginate or chitosan rely heavily on ionic or hydrogen-bonded networks, whereas proteins like gelatin or collagen contribute peptide motifs capable of temperature-dependent assembly or enzymatic remodeling [60]. Beyond composition, crosslinking density and mechanism exert significant influence on mechanical robustness, network porosity, degradation kinetics, and responsiveness. Covalent crosslinks typically enhance mechanical stability and strength but may reduce responsiveness and diminish dynamic adaptability, while supramolecular or ionic interactions offer tunable viscoelasticity and reversible responsiveness [61]. Importantly, the micro- and nano-scale architecture of the polymer network, including mesh size, heterogeneity, and the presence of secondary structural motifs, modulates diffusion of solutes, protein adsorption, and the rate at which stimuli propagate through the material. For natural hydrogels, subtle variations in chain arrangement or fibrillar organization can dramatically alter cell–matrix interactions or trigger sensitivity, as demonstrated in collagen-based systems where fibril density determines both stiffness and remodeling behavior [62]. Mechanical strength and fatigue resistance are crucial for tissue engineering applications where reinforcement with nanofillers or secondary polymers is often required [31]. In parallel, environmental conditions, such as pH, ionic strength, temperature, and oxidative state, directly shape network conformation by altering charge distribution, hydrogen bonding, or hydrophobic interactions. Hydrophilic-hydrophobic balance influences water retention and response speed. For instance, chitosan swelling is strongly pH-dependent due to protonation of amine groups, whereas gelatin undergoes sol–gel transitions near physiological temperatures [63]. Finally, biological interactions, including enzymatic degradation, protein corona formation, and cell-mediated mechanical loading, provide dynamic, context-specific forces that can strengthen, weaken, or adaptively reorganize the network over time. These interactions are particularly relevant in vivo, where the inflammatory microenvironment, characterized by specific pH changes, the presence of reactive oxygen species (ROS), and the secretion of various cytokines, can alter the hydrogel’s physical properties, thereby shaping therapeutic outcomes [64].

Regarding biocompatibility and degradation, natural polymers generally degrade enzymatically into safe byproducts, though batch variability and immune recognition remain challenges; consistency in molecular weight, purity, and gelation behavior is vital for clinical translation [35]. Together, these interconnected factors underscore that hydrogel performance cannot be attributed to any single variable but instead emerges from a synergistic balance between molecular composition, crosslinking chemistry, structural organization, and the external environment. Optimizing this interplay is crucial for rational design of next-generation, stimuli-responsive natural hydrogels with predictable and controllable behavior, tailored for specific biomedical tasks. In conclusion, natural polymer-based stimuli-sensitive hydrogels merge the biological affinity of nature-derived materials with adaptive functionality. The integration of multi-responsive systems, hybrid networks, and computational design is expected to further enhance control, reproducibility, and clinical applicability-setting the stage for the biomedical innovations explored in the following chapters.

## 3. Biomedical Applications

Advances in stimuli-responsive natural hydrogels are enabling therapies that adapt dynamically to the body’s physiological environment, driving a new generation of biomedical technologies. This chapter highlights how natural hydrogel systems are already reshaping key application areas by aligning material behavior with the body’s own regulatory mechanisms.

### 3.1. Controlled and Targeted Drug Delivery

Stimuli-sensitive natural hydrogels have matured into highly versatile platforms for controlled, on-demand release, and targeted drug delivery of therapeutic agents, cells or genetic payloads [9,28,65,66]. For instance, polysaccharide-based injectable hydrogels respond to pH, temperature or enzymatic triggers to release their load in a spatially and temporally controlled fashion [67]. In cancer therapy, hydrogel systems built on natural polymers can exploit the tumour microenvironment (e.g., acidic pH, elevated GSH levels) to effect triggered release of cytotoxics or immunotherapeutics. Studies have explored chitosan-based redox-responsive hydrogels for intracellular delivery of drugs in tumour cells [27].

Multi-stimuli systems (e.g., pH + temperature) increase selectivity and reduce off-target effects. Recent preclinical and early-phase clinical work has shown convincing advantages of such systems in addressing two persistent problems in drug delivery: (i) off-target toxicity and rapid systemic clearance, and (ii) failure to concentrate therapeutics at pathologic microenvironments. For oncology, for example, hydrogels designed to sense acidic pH and elevated GSH in tumours permit intratumoural or peri-tumoural retention and triggered intracellular release of chemotherapeutics or biologics, which reduces systemic exposure and enhances local potency [68]. In a recent translational study, in situ-forming HA-based hydrogels functionalized with redox-sensitive linkers showed controlled antibody release and prolonged local retention in tumour models, supporting the feasibility of HA hydrogels as local delivery depots for large biologics [69].

**Figure 5:**
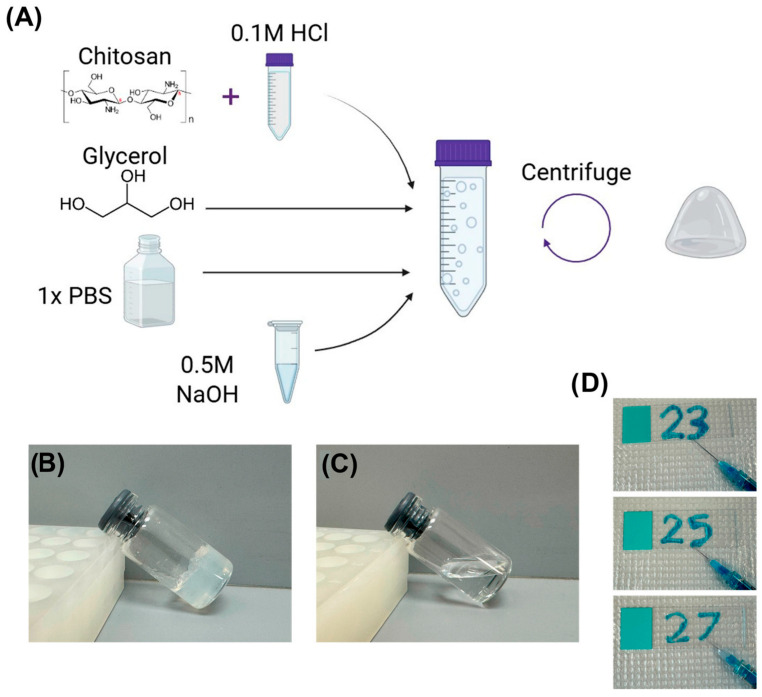
Formation and properties of chitosan–glycerol hydrogel. (**A**) Schematic of hydrogel preparation: chitosan dissolved in HCl-acidified phosphate-buffered saline, mixed with glycerol, neutralized with NaOH, then centrifuged and inverted to remove excess liquid. (**B**) The resulting hydrogel is non-flowable. (**C**) No gel forms without neutralization. (**D**) The hydrogel is injectable through 27-gauge needles; methylene blue was added for visualization. Reproduced from [71].

Thermogelling chitosan systems have been adapted for intratumoural, intravesical and local wound environments, and several groups have reported improved retention and therapeutic indices in animal models [28]. Tian et al. recently summarized clinical trial progress in chitosan hydrogel-based intravesical drug delivery systems for bladder cancer treatment [70]. Injectable thermogelling and enzyme-triggered chitosan/β-glycerophosphate hydrogels remain among the most clinically advanced natural hydrogel formats for localized therapy because they combine simple administration with gelation at physiological conditions. Chitosan-glycerol injectable hydrogels for intratumoral delivery of macromolecules have shown potential for future clinical use [71]. When glycerol was mixed with a chitosan solution prepared in dilute hydrochloric acid, a hydrogel formed after the mixture was neutralized with sodium hydroxide (Figure 5). The overall process for preparing the chitosan–glycerol hydrogel is illustrated in Figure 5A. Upon neutralization, an intermediate precipitate formed, but centrifugation produced a solid hydrogel (Figure 5B). Comparable results were obtained using acetic instead of hydrochloric acid. Neutralization was essential, as no gel formed without it (Figure 5C). The hydrogel exhibited viscoelastic behavior, recovered its shape after slight deformation, did not flow on a slide, and was injectable through 27-gauge needles (Figure 5D). Its pH was adjustable within the physiological range (6.8–7.5) by varying acid–base ratios.

Alginate- and HA-based hydrogels, owing to gentle ionic or enzymatic crosslinking and excellent cell compatibility, have been developed as depots for biologics and nucleic acid therapeutics [72,73]. Chemically modified alginates (e.g., oxidized or methacrylated derivatives) enable covalent tethering or programmable degradation, which facilitates controlled release kinetics appropriate for sustained local therapy and for combination regimens (e.g., chemo-immunotherapy) [74,75]. Clinically, several alginate-containing hydrogel products continue to be evaluated in trials for cardiac repair and cartilage regeneration, demonstrating that alginate platforms can be adapted as drug-eluting scaffolds [59]. These translational steps are important precursors to alginate use as delivery vehicles for anticancer agents or growth factors.

A particularly promising recent breakthrough is the integration of hydrogels with modalities for nucleic acid delivery (siRNA, mRNA). Stimuli-responsive hydrogels that protect labile RNA cargo and then release it in response to disease-relevant enzymatic or redox cues can unlock the full potential of RNA delivery, driving progress in precision medicine [76]. Primary reports have shown robust in vivo gene regulation in animal models. Reviews highlight hydrogel matrices engineered for spatiotemporal RNA delivery and propose paths toward clinical use, especially for local immunomodulation and regenerative cues where systemic administration is undesirable [77]. The modularity of natural hydrogels-allowing incorporation of lipid nanoparticles, polyplexes or peptide carriers-facilitates translation to RNA therapeutics while preserving biocompatibility [76,77].

Representative examples of biocompatible stimuli-sensitive natural hydrogels engineered for controlled and targeted drug delivery are presented in Table 4. Each system leverages specific physicochemical triggers (such as pH, redox potential, enzymatic activity, temperature, or ionic exchange) to modulate polymer network structure and thereby regulate payload release. Recent preclinical and translational studies (2023–2025) demonstrate clinical promise for localized cancer therapy, wound healing, and regenerative medicine [26,27,40,59,65,69,75,76,77,78,79,80]. Key design criteria for drug delivery include: the responsiveness threshold (e.g., pH drop or enzyme up-regulation), swelling/deswelling kinetics, mesh-size tuning to regulate diffusion, and degradation rate to ensure safe clearance. While synthetic hydrogels have dominated historically, the push to natural-polymer platforms addresses translational barriers such as immunogenicity and regulatory acceptability [9]. Nevertheless, challenges remain; we will review them in a separate chapter (e.g., achieving burst-free release profiles, ensuring stability in physiological fluids, avoiding premature release during circulation, and scaling production for clinical use).

### 3.2. Tissue Engineering and Regenerative Medicine

Natural stimuli-sensitive hydrogels also play a pivotal role in tissue engineering and regenerative medicine by serving as a scaffold because the hydrated, ECM-like network of natural polymers offers intrinsic bioactivity (cell-adhesive motifs, enzymatic degradability) that supports cell attachment, migration, and differentiation, while stimuli-responsive chemistries permit on-demand modulation of mechanical properties, porosity and degradation to match tissue repair timelines [81,82]. A major clinical focus has been cartilage repair, where injectable, in situ gelling hydrogels that respond to mechanical load, enzymatic activity or ionic changes have been used as cell carriers or growth-factor depots to promote matrix deposition and integration for focal defects [78,83,84]. The ability to tune hydrogel stiffness via stimuli (e.g., temperature or ion-exchange) is particularly valuable since stem cell fate is sensitive to mechanical stress. Several recent translational efforts report encouraging safety and early efficacy signals in clinical trials for cartilage regeneration [85]. Preclinical work and clinical case series indicate that hydrogels supporting chondrogenesis and controlled degradation can fill irregular defects and promote hyaline-like tissue formation, reducing donor-site morbidity compared with autografts [86,87].

Injectable hydrogels that gel in situ at physiological temperature (thermoresponsive) permit minimally invasive delivery of cell-hydrogel constructs into irregular defects. Once cured, these scaffolds can degrade as the tissue regenerates, avoiding the need for surgical removal [26]. Emerging strategies include 3D bioprinting of stimuli-sensitive natural hydrogels to fabricate complex architectures with spatial variation in responsiveness and biological cues. Integrating growth factor release triggered by local enzymes or redox conditions further enhances control over the regeneration process. However, the translation of these systems to large-scale, load-bearing tissue repair remains a challenge, largely due to limitations in mechanical strength, vascularisation, and long-term integration [35].

**Figure 6:**
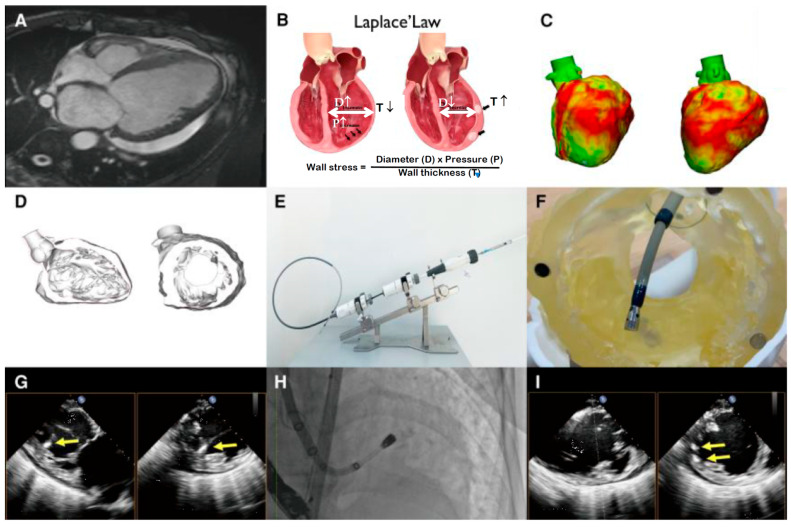
Transcatheter endocardial alginate hydrogel implantation (TEAi) in a patient with dilated cardiomyopathy. (**A**) MRI showing LV enlargement. (**B**) Concept of LV wall augmentation with alginate hydrogel based on Laplace’s law. (**C**,**D**) 3D printing used to assess wall thickness and simulate the procedure. (**E**) Dual-lumen catheter for alginate hydrogel delivery via an 18-F femoral approach. (**F**–**H**) Catheter positioned at the LV endocardium (arrow in **G**) contrast confirmed no leakage or perforation. (**I**) Alginate hydrogel (arrows) injected at 10 mid-LV wall sites. At 6 months, MRI showed improved left ventricular ejection fraction (22%) and reduced LV volumes, with clinical improvement. Reproduced from [89].

Cardiac repair is another area with tangible translational progress [88]. Alginate-based injectable hydrogels have been explored as intramyocardial bulking agents to prevent adverse remodeling after myocardial infarction. Notably, a first-in-human transcatheter endocardial alginate hydrogel implantation study reported feasibility and safety in heart failure patients, demonstrating that a locally delivered hydrogel can be delivered via catheter and remain in place to support ventricular geometry [89]. A 55-year-old man with dilated cardiomyopathy and decompensated heart failure underwent evaluation for transcatheter endocardial alginate hydrogel implantation (TEAi) in a clinical trial (NCT04781660), presented in Figure 6. Baseline magnetic resonance imaging (MRI) showed a markedly enlarged left ventricle (LV) with severely reduced ejection fraction. The TEAi procedure, guided by fluoroscopy and transesophageal echocardiography, involved endocardial injections of alginate hydrogel at 10 mid-LV sites (total 3 mL) to thicken the ventricular wall and reduce wall stress based on Laplace’s law. Six months later, the patient’s symptoms improved, and MRI demonstrated better left ventricular ejection fraction function and smaller LV volumes, supporting the safety and feasibility of TEAi for advanced heart failure.

Subsequent preclinical advances, using alginate hydrogels loaded with pro-regenerative mediators such as annexin A1 or bone marrow cells, have shown improved cardiac function and attenuated fibrosis in animal models, supporting ongoing translational development [90].

Bone and osteochondral regeneration benefit from stimuli-sensitive hydrogels that couple mechanical reinforcement with controlled release of osteoinductive factors. Hybrid hydrogels that incorporate cellulose nanocrystals, bioactive glass or calcium phosphate nanoparticles improve stiffness while serving as reservoirs for BMP-2 or other growth factors released in response to enzymatic cues or pH changes during remodeling [40]. Recent clinical reports and animal studies support the use of composite alginate/gelatin and HA-based scaffolds for small bone defects and spinal fusion adjuncts, although large, randomized trials remain limited [91,92].

Skin and soft tissue regeneration using stimuli-sensitive hydrogels have progressed rapidly toward clinical translation because of the comparatively accessible treatment site and lower mechanical demands. Chitosan- and HA-based dressings that release antimicrobials or growth factors in response to wound pH or protease activity improve healing in chronic wounds in preclinical and early-phase clinical studies [27,93]. A recent pilot clinical study used injectable chitosan hydrogel particles as nasal packing after endoscopic sinus surgery, demonstrating good hemostasis, improved patient comfort and favourable wound healing metrics, an example of direct clinical adoption of a stimuli-tunable natural hydrogel formulation [79].

Cell delivery and 3D bioprinting represent convergent translational advances. Stimuli-adaptable hydrogels that modulate stiffness or present degradable cell-adhesive properties enable encapsulated stem cells or progenitors to survive, spread and differentiate after implantation. Progress in bioink formulation, combining natural polymers with reversible crosslinks or shear-thinning behavior, has enabled high-fidelity bioprinting of cartilage, vascular and skin constructs [94,95,96]. Although most bioprinted constructs are still in preclinical stages, a growing number of clinical case reports and registered early-phase trials indicate movement toward human application, particularly in reconstructive surgery and cartilage regeneration [97]. Recent breakthroughs that accelerate clinical translation include: (i) dual-crosslinked hybrid hydrogels that offer rapid in-situ gelation yet long-term mechanical integrity [98]; (ii) enzyme-sensitive peptide linkers tailored to specific tissue protease profiles for temporally staged degradation; and (iii) integration of extracellular vesicles or growth factor-loaded nanoparticles within hydrogels to provide sustained paracrine signaling [40,82]. These strategies improve functional regeneration in multiple models and provide modularity for clinical product design.

### 3.3. Wound Healing

Chronic and acute skin wounds present unmet clinical needs that demand dressings and scaffolds capable of managing infection, modulating inflammation, and supporting tissue regeneration. Stimuli-sensitive natural hydrogels address these requirements by combining moist wound environment, conformability to irregular wound beds and protection from contaminants with the capacity to incorporate antimicrobials or growth factors. When made stimuli-sensitive, these hydrogels adapt dynamically to the healing environment, adding on-demand functionality. For example, pH-responsive systems can release antimicrobials when the wound becomes acidic during infection; enzyme-responsive hydrogels can degrade in response to proteases present in chronic wounds [46]. Hydrogels with redox responsiveness can deliver antioxidants under oxidative stress typical of chronic wounds, enhancing healing and reducing inflammation. Such behaviours have been reported in advanced hydrogel dressings that combine natural biopolymers with antimicrobial nanoparticles and stimuli-triggered release [27]. The conformability and injectability of natural hydrogels make them suitable for burn sites, diabetic foot ulcers and surgical incisions. Challenges include maintaining mechanical stability on mobile skin regions, ensuring adhesion, and managing degradation products safely over extended treatment periods.

**Figure 7:**
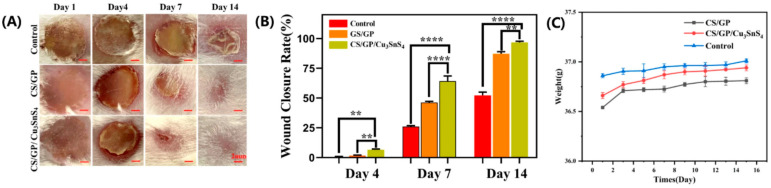
Evaluation of wound healing and body weight in mice. (**A**) representative images showing wound appearance over time, (**B**) wound closure rate, asterisks (**, ****) denote higher levels of statistical significance, and (**C**) monitoring of body weight changes during the healing process. Reproduced from [101].

Chitosan-based hydrogels remain among the most studied natural platforms for wound care owing to inherent hemostatic, mucoadhesive and antimicrobial properties, plus facile chemical modification. Recent work has produced self-healing chitosan hydrogels that restore network integrity after mechanical disruption, improving conformability over mobile skin regions and prolonging functional life on wounds [99,100]. Chitosan matrices loaded with antimicrobial nanoparticles (e.g., Cu- or Ag-containing nanostructures) or antimicrobial peptides demonstrate potent in vitro bactericidal activity and accelerated closure in animal infection models [101,102]. It is known that burns remain a major global health problem, often complicated by infections and poor healing. To address this, a chitosan-based hydrogel loaded with copper-tin-sulfur nanoparticles was developed as a novel burn dressing. The prepared hydrogel showed strong antibacterial activity (>95% against *S. aureus* and *E. coli* within 4 h), good biocompatibility, and significantly enhanced wound healing in vivo (Figure 7).

Moreover, clinical translation is beginning: chitosan formulations have been repurposed as nasal packing and surgical dressings in pilot human studies that document safety and improved wound metrics-evidence that establishes regulatory and manufacturing pathways for more advanced, stimuli-tuned chitosan dressings [79,103].

HA-based hydrogels play a complementary role because HA is a native ECM glycosaminoglycan that modulates cell migration, angiogenesis and inflammation. Functionalized HA hydrogels incorporating redox- or enzyme-labile linkages enable controlled degradation and release of therapeutics in response to the oxidative and protease-rich environment of chronic wounds. Redox-sensitive HA gels have demonstrated accelerated re-epithelialization and reduced oxidative stress in diabetic wound models [104,105]. Recent preclinical HA constructs combining photothermal or antimicrobial modalities also show promise for infected or non-healing wounds, although human data remain limited and warrant rigorous trials [106].

Alginate and alginate-composite dressings are well-established clinically for exudate management, and recent developments extend alginate’s role into stimuli-responsive depots. Ionically crosslinked alginate (Ca^2+^) remains attractive for easy fabrication and high absorptivity; incorporation of zinc or other bioactive ions can add antimicrobial or pro-healing activity. Meta-analyses and systematic reviews published in 2023–2025 indicate that hydrogel and alginate dressings can improve healing rates in diabetes and venous leg ulcers relative to basic dressings in selected trials, though effect sizes vary and high-quality randomized data are still sporadic [103,104]. These clinical data underscore both the potential and the need for standardized endpoints in hydrogel wound trials [107,108].

A recent breakthrough is the application of redox- and enzyme-responsive hydrogel platforms that actively modulate pathologic wound microenvironments. Chronic wounds are characterized by elevated ROS and protease levels that impede healing; responsive hydrogels that scavenge ROS while releasing growth factors when protease activity rises can normalize the milieu and accelerate repair. Genetically encoded or protein-based redox-sensitive hydrogels have shown marked efficacy in diabetic wound models by upregulating collagen I and promoting re-epithelialization [105,109]. Translationally, some formulations that combine ROS scavenging with antimicrobial delivery are approaching GLP toxicology and large-animal evaluation, positioning them for early clinical testing [49,105].

Antimicrobial strategies integrated within hydrogels have also advanced. Beyond conventional antibiotics, antimicrobial peptides, metal-ion complexes, and photothermal/photodynamic agents are being embedded into natural polymer networks to create multifunctional dressings that both reduce bioburden and actively promote regeneration [102,110]. Importantly, several recent reviews emphasize that antimicrobial peptides and nanoparticle strategies can modulate inflammation and angiogenesis in ways that benefit healing beyond simple bacterial kill rates-an observation key to designing next-generation dressings [102,110].

Recent progress in stimuli-responsive natural hydrogels for wound healing has produced a diverse landscape of smart dressings, yet their relative advantages can be more clearly understood when evaluated against key performance domains such as cost-effectiveness, cooling function, anti-inflammatory efficacy, multifunctionality, and regenerative support. While earlier-generation hydrogels successfully offered moisture retention and biocompatibility, the latest innovations demonstrate significant leaps in functional breadth, therapeutic impact, and clinical translation potential. A notable advancement is the development of low-cost natural-polymer dressings without sacrificing responsiveness or bioactivity. For instance, a recent study [111] introduced a biopolymer-based anti-inflammatory hydrogel patch designed for affordable large-scale production. Its formulation relies on abundant natural polysaccharides and mild gelation conditions, marking a significant improvement over earlier systems that required costly crosslinkers or complex fabrication steps. This represents a tangible shift toward economic feasibility, particularly for chronic-wound management in low-resource settings—an aspect often overlooked in the hydrogel literature. Several next-generation hydrogels incorporate endothermic swelling or rapid water-evaporation dynamics to provide cooling effects at the wound interface. Compared to conventional hydrogels that primarily serve as passive moisture barriers, modern formulations actively reduce local temperature and inflammation, improving patient comfort. This functional enhancement is particularly valuable for burns and acute inflammatory lesions and demonstrates how polymer composition and network hydration dynamics can be tuned to deliver clinically meaningful cooling without external energy input. Improvements in bioactivity are among the most impactful advancements. Modern hydrogel dressing exhibit robust inhibition of inflammatory cytokine expression, outperforming earlier chitosan or alginate hydrogels that primarily relied on intrinsic polymer properties. By incorporating anti-inflammatory phytochemicals or nanoparticles into natural matrices, these new systems achieve dual-function action: protecting the wound while modulating the immune microenvironment. This directly addresses challenges noted in early clinical trials, where hydrogels often enhanced healing but lacked targeted control over inflammation. A major functional leap is reflected in recent multifunctional hydrogel dressings with real-time sensing. A notable example reported recently [112] combines a natural hydrogel matrix with flexible microelectronic sensors capable of detecting wound pH, temperature, and biomarker flux. This represents a substantial evolution from previous “one-function” dressings by enabling closed-loop monitoring and potentially automated therapeutic release. Such platforms directly respond to the clinical need for continuous wound surveillance, decreasing physician burden and enabling earlier detection of infection or decompensation.

Another meaningful advancement is the design of ECM-inspired bioprinted, natural-polymer hydrogel scaffolds engineered with microchannel architectures mimicking native vascular network that actively promote neovascularization [113]. This structural innovation leads to significantly enhanced angiogenesis compared to earlier uniform hydrogels, representing a true generational improvement in regenerative potential. In contrast to previous hydrogels that merely created a favorable moist environment, these biomimetic constructs directly instruct cell organization and vascular ingrowth.

In contrast to earlier hydrogel dressings that provided moisture retention and basic protection, the studies highlighted above collectively demonstrate a transformative shift toward affordability and scalable manufacturing, enabling broader clinical access; advanced cooling and pain relief; targeted anti-inflammatory and antimicrobial action, improving outcomes in chronic wounds; integrated sensing and feedback mechanisms, supporting next-generation digital wound care, and bioinspired architectures supporting robust angiogenesis, accelerating regenerative healing.

Taken together, these advancements illustrate that the field is moving beyond incremental improvements and toward multi-functional, clinically oriented, and economically viable hydrogel systems capable of addressing longstanding unmet needs in wound management. Over the past five years, this class of materials has advanced from proof-of-concept constructs to a growing number of preclinical demonstrations and a steady flow of early-phase trials and pilot clinical studies evaluating hydrogel dressings for chronic wounds, such as diabetic foot and venous leg ulcers, and surgical sites. Systematic meta-analysis of trials for diabetes foot ulcers up to 2024 shows improved healing metrics, reduced dressing changes, and acceptable safety compared with conventional care, although heterogeneity of studies and small sample sizes temper conclusions [59,102,107,114,115]. Despite encouraging progress, several clinical and translational challenges remain. First, robust evidence in large randomized controlled trials is limited; many published studies are small or lack rigorous blinding, and outcome measures differ across trials. Second, manufacturing and regulatory hurdles for multifunctional hydrogels, especially those combining biologics, antimicrobial peptides or nanoparticles, are nontrivial; establishing sterility, storage stability, and GMP-grade sourcing of natural polymers is essential [59]. Third, patient-level factors (comorbidities, blood supply, infection status) strongly influence responses and must be integrated into trial design and patient selection. Addressing these constraints requires coordinated translational programs that combine materials science with clinical expertise and regulatory planning, pointing to the need for larger, well-controlled trials with standardized endpoints.

**Figure 8:**
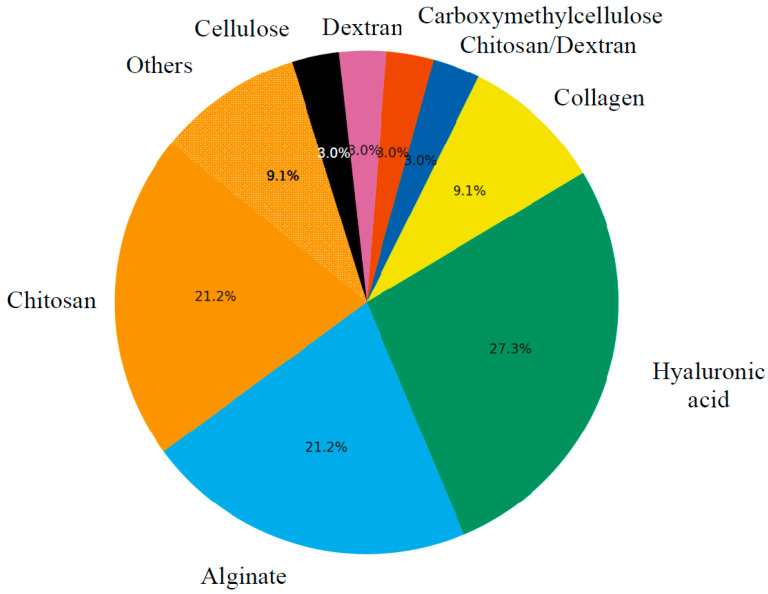
Distribution of registered trials by natural hydrogel system (from ClinicalTrials.gov).

Table 5 is a representative table of recent clinical trials and primary/translational reports of stimuli-sensitive natural hydrogel systems across the main biomedical application areas (drug delivery/local depots, tissue engineering, wound healing, ophthalmology, endoscopy/interventional uses, aesthetic/soft-tissue), listing each trial’s NCT identifier as recorded on ClinicalTrials.gov trial page, for fact-checking, hydrogel type (natural polymer), indication, phase and status. This table below is representative (captures major trials across the range of applications) rather than exhaustively listing every ongoing or completed trial worldwide. The table focuses on trials using natural-polymer hydrogels (chitosan, alginate, HA, gelatin/collagen or composites). It includes devices/products that use ionic/thermogelation or enzymatic/biocompatible gelation, even if the trial record does not explicitly label the system as “stimuli-sensitive.” Many clinically tested hydrogel systems employ in situ gelation (thermo/ionically triggered) or biodegradable chemistries relevant to stimuli-responsive design. Trial status is reported as listed on ClinicalTrials.gov. Some entries are historic or early feasibility studies (e.g., NCT00004487, IK-5001) that demonstrate prior clinical translation of natural hydrogel concepts; others are ongoing or more recent (2022–2025) and reflect the current translational pipeline. The trial ID allows one to verify up-to-date status and details (Figure 8).

### 3.4. Biosensing and Diagnostic Applications

Beyond therapy and regeneration, stimuli-sensitive hydrogels have found growing use in biosensing and diagnostics. Hydrogel-based biosensors are an emerging class of diagnostic interfaces that combine the soft, hydrated, and biofriendly environment of polymer networks with transduction mechanisms that convert biochemical or physical changes into measurable signals. Natural-polymer hydrogels are particularly attractive as sensing matrices because they provide tissue-like mechanical compliance, facile chemical functionalization, and intrinsic biomolecular affinity, attributes that support minimally invasive, continuous, or point-of-care diagnostics [116,117], and can be engineered to change colour, size, optical or electrical properties in response to specific biomolecular triggers (e.g., glucose, enzymes, pH). For example, aptamer-functionalised hydrogels collapse or swell upon binding target molecules, enabling biomarker detection via optical readouts [118]. Such platforms are appealing in point-of-care diagnostics: the hydrogel matrix maintains a biological interface, while the stimuli-response allows signal amplification. Although many reporting studies have used synthetic polymers, prototypes using natural-polymer hydrogels (e.g., HA or cellulose) are emerging, offering enhanced biocompatibility for in vivo sensing. Design considerations include specificity of the stimulus, rate of response, reversible vs. irreversible change, and integration with transducers or electronics. Challenges lie in achieving robust, repeatable responses in complex physiological fluids, and integrating natural hydrogels into compact sensor devices.

Design strategies for hydrogel biosensors typically exploit three roles for the hydrogel: (i) a selective recognition layer (immobilized aptamers, enzymes, antibodies or molecularly imprinted sites), (ii) a stimulus-transducing matrix (where swelling, optical index, conductivity, or mechanical properties change on target binding), and (iii) an interface to a readout module (optical, electrochemical, piezoelectric or wireless electronics) [117,119]. By integrating natural hydrogels with conductive fillers (e.g., carbon nanomaterials, metal nanowires) or photonic structures, sensors can achieve high sensitivity while maintaining biocompatibility for in vivo or on-skin applications [119,120]. Recent breakthroughs span wearable devices (sweat and interstitial analytes), tear-based diagnostics (smart contact lenses), implantable biosensing arrays, and point-of-care visual/colourimetric hydrogel strips for infection detection. In wearable sweat sensors, conductive or ionically responsive hydrogels serve as intimate interfaces that sample minute sweat volumes and transduce ionic/biomarker concentrations into electrical signals; recent work demonstrates continuous sweat induction (iontophoresis) coupled with agarose/polysaccharide hydrogel collectors and integrated electronics for robust on-body monitoring [121]. These platforms are progressing rapidly toward human validation for hydration, electrolyte balance and metabolite monitoring.

Tear-based hydrogel sensors-most notably smart contact lenses-represent one of the most visible translational pathways for hydrogel diagnostics because the eye offers a relatively accessible, conformal biofluid reservoir. Over the past five years, teams have advanced soft, wireless contact lenses that integrate hydrogel matrices with microelectronic sensors for glucose and intraocular pressure monitoring; importantly, recent human-relevant studies have evaluated correlations between tear and blood glucose using soft lens platforms and reported improved concordance and wearable performance metrics [122,123]. Such studies provide encouraging evidence that hydrogel contact-lens sensors are approaching clinical practicality, although broad regulatory approval remains pending.

**Figure 9:**
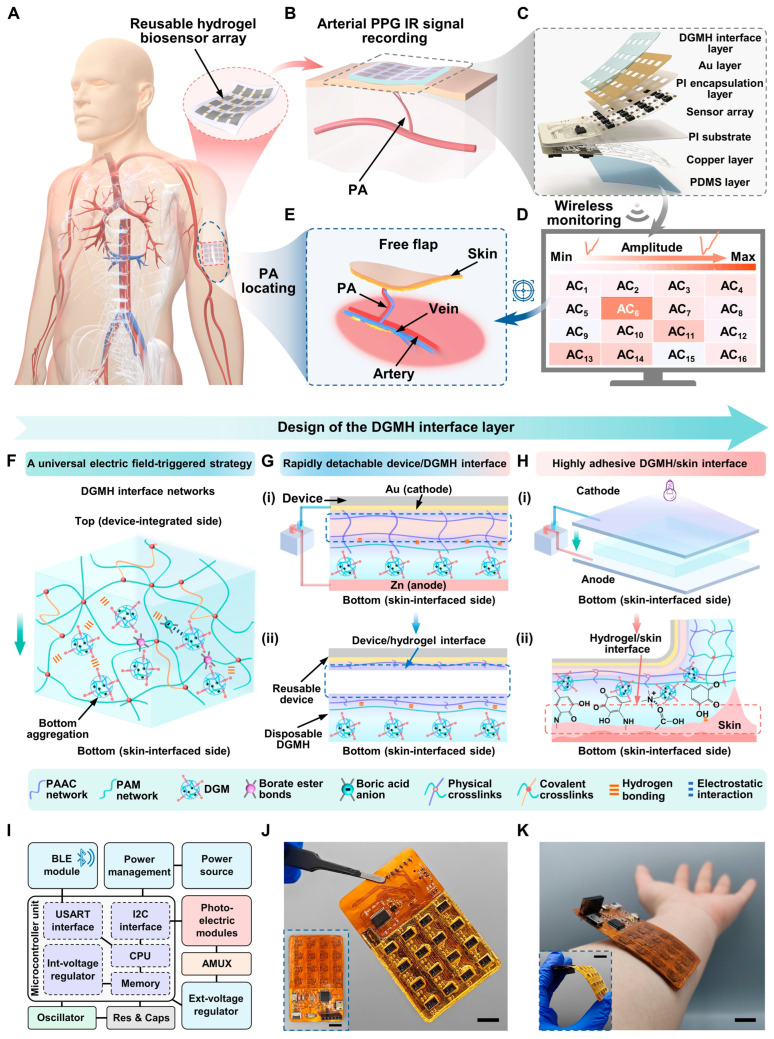
Design and structure of the reusable hydrogel biosensor array. (**A**,**B**) Schematic illustration of the biosensor array applied to a free flap donor site and its use in locating perforating arteries (PAs) via photoplethysmography (PPG) signals. (**C**–**E**) Structural overview and principle of PA localization and flap guidance. (**F**–**H**) Design and mechanism of the gallic acid modified hydrogel microspheres (DGMH) interface layers enabling detachable yet adhesive hydrogel–device–skin integration. (**I**) Block diagram of key electronic components. (**J**,**K**) Photographs of the biosensor array and hydrogel layer integrated with the device on the arm. Scale bars: 10 mm. Reproduced from [120].

In the implantable domain, hydrogel biointerfaces are being combined with microelectrode arrays and reusable biosensor platforms to improve signal fidelity and biocompatibility. A 2025 reusable hydrogel biosensor array demonstrated electrically coupled mapping capability for flap perfusion assessment and was tested in clinical scenarios to localize perforator arteries, illustrating that hydrogel-based implantable or percutaneous sensors can reach translational evaluation [120]. These systems leverage hydrogel mechanics to minimize foreign-body reaction while enabling stable electrical coupling. Figure 9 shows the reusable hydrogel biosensor array for precise and efficient perforating artery (PA) localization. The system integrates a flexible multichannel PPG (photoplethysmography) circuit with a removable interface layer of gallic acid-modified hydrogel microspheres (DGMH). A ferric ion-mediated coordination bond enables strong device/hydrogel attachment, while a Zn-Au electric field triggers rapid detachment (within 8 s), allowing easy replacement and reuse to prevent cross-infection. The DGMH layer, fabricated under a +2 V electric field, exhibits improved adhesion (>7-fold), elasticity, and antibacterial properties, ensuring stable skin contact and high-quality signal capture. Clinically, the array enables precise, noninvasive, and operator-independent perforating arteries (PA) mapping with superior portability compared to computed tomography angiography or acoustic Doppler sonography. This electric field-triggered interface strategy provides a general approach for creating reusable, high-performance flexible bioelectronic systems [120].

Hydrogel-based bacterial infection diagnostics have also advanced markedly. Because infection alters local microenvironmental cues (pH, protease activity, metabolic byproducts), responsive hydrogels that change color, optical scatter, or electrical impedance upon bacterial growth enable rapid visual or instrumented detection. Recent devices embed nanozymes or enzyme-responsive chromogenic elements into HA or alginate matrices to generate dual-mode readouts (colour + electrochemical) with limits of detection suitable for wound or urine screening; several prototypes are now in preclinical large-animal validation and some are entering GLP toxicology pipelines preparatory to first-in-human studies [124,125].

From an analytical performance perspective, hydrogel sensors bring unique advantages: tunable mesh size controls analyte partitioning (enhancing selectivity), incorporation of affinity motifs improves specificity, and the hydrated milieu preserves bioactivity of immobilized enzymes or aptamers-crucial for prolonged, reliable measurement [108,109]. Hybridizing hydrogels with plasmonic or photonic nanostructures enables ultrasensitive optical readouts (surface-enhanced Raman scattering, localized surface plasmon resonance (LSPR) shifts) while conductive hydrogel composites confer low-impedance electrical contact for electrophysiological or electrochemical sensing [120,124].

#### 3.4.1. Advances in Skin-Interfaced Bioelectronic Hydrogel Sensors

While stimuli-responsive natural hydrogels have long served as soft, biocompatible platforms for biosensing, recent progress in skin-interfaced bioelectronics has fundamentally expanded their capabilities, enabling high-fidelity electrophysiological monitoring, long-term wearability, and seamless integration with human biomechanics. Conventional hydrogel electrodes often suffer from dehydration, poor mechanical robustness, and limited reusability, which restrict their clinical applicability. In contrast, next-generation skin-mounted hydrogel biointerfaces combine conductivity, bioadhesion, ultrasoft mechanics, and printability, representing a substantial functional leap [126].

One of the most important developments is the emergence of 3D-printable and bioadhesive conductive hydrogels that maintain conformal skin contact under movement. Recent examples include supramolecular and polymer–nanocomposite formulations that permit high-resolution patterning of conductive pathways directly onto soft substrates [127]. Similar approaches using dynamic covalent networks and nanofiber reinforcement have provided printable hydrogels with superior toughness and long-term adhesion [128]. These materials overcome limitations of earlier hydrogel electrodes by ensuring mechanical durability, humidity resistance, and minimal interfacial impedance drift, which are essential for continuous monitoring in ambulatory settings. Another significant advance involves the development of reusable hydrogel-based platforms for high-resolution electromyography (EMG). For example, one adhesive yet non-irritating hydrogel EMG interface demonstrated stable signal acquisition over many cycles of attachment and detachment. This reusability not only reduces patient burden and cost but also represents a clear improvement over single-use hydrogel electrodes commonly deployed in clinical settings [129]. Further innovations include highly stretchable and conformable hydrogel arrays [130], offering precise mapping of muscle activation patterns during complex movements. These systems bridge the gap between rigid clinical electrodes and highly deformable skin surfaces, enabling high-density, artifact-reduced EMG in real time. The field has also benefited from pioneering work on ultrasoft conductive hydrogels with mechanical moduli that closely match human epidermis, improving comfort and reducing motion artifacts. For example, supramolecular hydrogel interfaces [131] employ reversible host–guest interactions to maintain conductivity and adhesion under strain, providing an exceptional skin-like feel. Similarly, ultralow-modulus conductive hydrogels are capable of capturing faint electrophysiological signals with signal-to-noise ratios previously unachievable in natural hydrogel systems [132]. These interfaces exemplify how precision molecular engineering can tune viscoelastic properties to match the mechanical characteristics of skin, enabling high-fidelity physiological tracking even during vigorous motion.

Collectively, these emerging technologies demonstrate that skin-interfaced hydrogel bioelectronics are moving far beyond conventional sensing toward fully integrated, multimodal, and reusable interfaces capable of seamless human–device interaction. By combining high conductivity, robust adhesion, mechanical softness, printability, and durability, these advances address major limitations outlined in Section 5, particularly mechanical fragility, dehydration, and poor reproducibility, while charting a clear path toward clinically viable wearable diagnostic systems.

#### 3.4.2. Clinical Translation

Clinical translation and human validation are accelerating but remain heterogeneous by application. Tear-lens and wearable sweat platforms have progressed furthest in human testing: several studies report in-human correlation and wearability metrics, and larger clinical validation studies are ongoing to calibrate analyte correlations and long-term tolerability [122,133]. Implantable hydrogel sensor arrays and point-of-care infection tests are primarily in advanced preclinical or early clinical feasibility phases, with a handful progressing to small human pilot studies for device localization or wound monitoring [120,124]. Overall, the translational landscape now includes bench-to-bedside demonstrations of function and safety for soft hydrogel sensors, but widespread regulatory clearance for diagnostic labeling will require multicentre clinical studies with standardized endpoints.

Key challenges for clinical adoption include: (i) calibration and drift-hydrogel properties (swelling, enzyme activity) can evolve during wear, necessitating robust calibration strategies or self-referencing designs; (ii) biofouling and longevity, which demand anti-fouling chemistries or periodic replacement for chronic monitoring; (iii) integration with electronics while preserving sterilizability and biocompatibility; and (iv) regulatory and data-quality standards for continuous biochemical monitoring [134,135]. Addressing these requires cross-disciplinary engineering, robust manufacturing, and clinical studies that quantify device accuracy under real-world conditions.

Emerging directions likely to accelerate clinical impact include (a) closed-loop therapeutic systems in which hydrogel sensors feed signals to drug-eluting hydrogels (theranostic patches), (b) modular hydrogel sensor arrays that multiplex analytes for richer physiological readouts (e.g., electrolytes + metabolites + biomarkers), and (c) AI-assisted calibration and signal processing to translate noisy biosensor outputs into clinically actionable metrics [134,135]. These approaches promise to move hydrogels from single-analyte demonstrators toward integrated diagnostic platforms for chronic disease management and perioperative monitoring.

Hydrogel-based biosensors leverage the unique physicochemical match between soft polymer networks and biological tissues to enable minimally invasive, wearable and implantable diagnostics. Recent progress, from human tear-lens correlation studies to implantable hydrogel sensor arrays tested in clinical contexts, indicates that the field is transitioning from laboratory prototypes to translational devices. The next 3–5 years will be decisive: success will hinge on robust human trials demonstrating accuracy, durability and clinical utility, and on regulatory pathways that can accommodate continuous, biointegrated diagnostics built from natural hydrogel platforms. Table 6 summarizes representative hydrogel sensor prototypes and their human-testing/clinical status. Each row lists the device or prototype, the primary polymer matrix, the target analyte or sensing function, the study type, and the clinical stage/status.

### 3.5. Injectable and In Situ-Forming Hydrogel Systems

**Figure 10:**
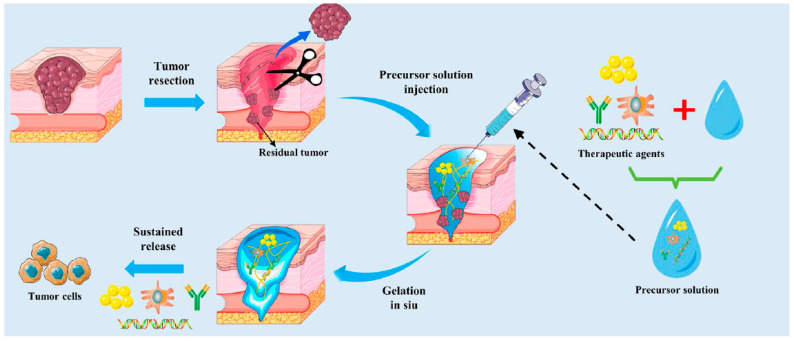
Schematic illustration of an injectable in situ-forming hydrogel for prevention of postoperative tumor recurrence. Reproduced from [93].

Injectable and in situ-forming hydrogels constitute one of the most clinically attractive branches of stimuli-sensitive biomaterials because they enable minimally invasive delivery, conformal filling of irregular defects, local depot formation and temporal control of mechanical and biochemical cues after administration [93]. These systems are typically designed as low-viscosity precursors that gel in response to physiological triggers (temperature, ionic strength, pH, enzymes, or redox conditions) or to mild external inputs (light, ultrasound), producing a 3D hydrated scaffold at the target site without the need for open surgery (Figure 10) [93].

Natural polymers dominate the injectable hydrogel landscape because of their inherent biocompatibility and degradability. Stimuli responsiveness enhances these platforms: an in situ hydrogel may undergo further changes after gelation (e.g., degrade in response to enzymes, swell in response to local pH changes), thereby tailoring its behaviour to the local microenvironment. These features are increasingly adopted in treatments for myocardial infarction, spinal cord injury, cartilage defects and localized tumour therapy. The key constraints remain gelation kinetics (must be rapid but controlled), injectability, biocompatibility of gelation mechanisms, and post-gel mechanical stability. Scaling these systems for clinical use and sterilisation remain major translation hurdles.

#### 3.5.1. Materials and Gelation Mechanisms

Chitosan/β-glycerophosphate (β-GP) systems are archetypal thermogelling natural hydrogels: precooled chitosan solutions mixed with β-GP remain flowable at room temperature and undergo rapid sol→gel transitions at ~37 °C, enabling syringe delivery followed by depot formation [151]. Chemical tuning (molecular weight, degree of deacetylation, β-GP ratio) allows control of gelation time, mechanical modulus and degradation kinetics [151,152].

Ionic crosslinking (alginate/Ca^2+^) provides another clinically relevant, mild gelation route: injectable alginate solutions are crosslinked after administration via multivalent cations to form hydrogels suitable for cell encapsulation or depot delivery [153]. Enzymatic crosslinking (e.g., transglutaminase, horseradish peroxidase) and click/photochemical reactions enable covalent network formation under cytocompatible conditions and are widely used when longer-term mechanical stability is required [154]. Redox- or protease-labile linkers are commonly incorporated to achieve stimulus-triggered degradation and release in diseased microenvironments [155].

#### 3.5.2. Key Translational and Clinical Milestones

Several injectable natural-polymer hydrogels have progressed to human trials or regulatory milestones, illustrating translational feasibility. Alginate injected into infarcted myocardium was among the first hydrogel strategies translated to humans. Early first-in-man studies demonstrated that intracoronary or intramyocardial alginate injections are feasible and well tolerated, with signals of improved ventricular geometry and exercise tolerance supporting further trials [153,156,157]. The IK-5001 program (registered as NCT01226563 [158]) and subsequent cardiac alginate studies represent an important proof-of-concept for injectable biomaterial therapy in heart failure.

**Figure 11:**
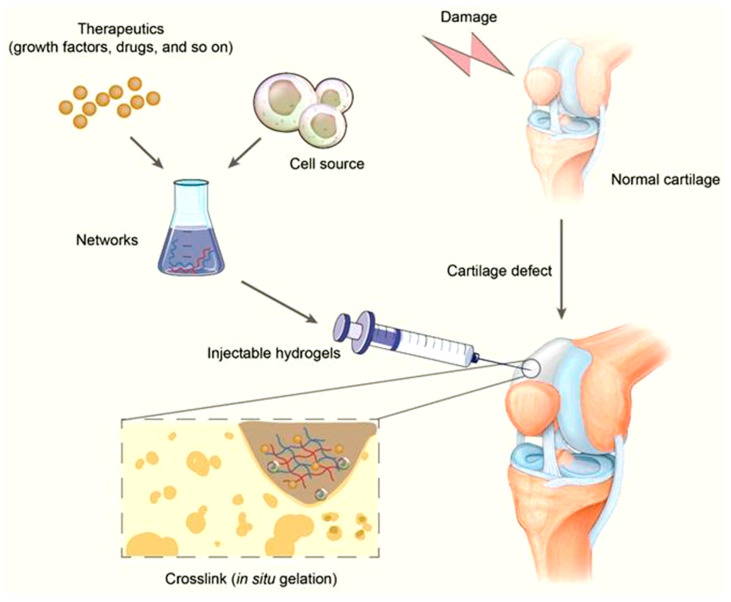
Schematic diagram of the application of injectable hydrogels for cartilage repair. Reproduced from [159].

Injectable cartilage hydrogels are rapidly moving into clinical evaluation [80,159]: reflecting robust translational momentum for minimally invasive cartilage restoration (Figure 11).

These studies target focal cartilage defects where conformal in situ gels can act as cell- or growth-factor carriers and structural fill. Injectable chitosan thermogels: chitosan/β-GP formulations have been evaluated in a range of translational contexts because of mild gelation and mucoadhesive properties. Clinical and pilot uses -ranging from topical mucosal gels to hemostatic dressings and postoperative packing- demonstrate acceptable safety and functional benefit, establishing manufacturing and sterilization pathways for more complex drug-eluting chitosan hydrogels [160,161]. Beyond these specific products, numerous in situ-forming hyaluronic-acid (HA) derivatives and composite hydrogels have shown promising preclinical efficacy in cartilage and soft-tissue repair and are entering first-in-human or early feasibility studies for regenerative indications [155,162].

Several recent technological advances have accelerated the clinical readiness of injectable hydrogels: (i) dual-crosslinking and hybrid networks—combining a rapid physical crosslink (for immediate handling and shape retention) with a slower covalent crosslink (for long-term stability)—yields injectables that gel quickly yet achieve higher mechanical strength and controlled degradation, an approach increasingly adopted in translational formulations [69]; (ii) payload-compatible manufacturing and sterilization: improved sterilization approaches (e.g., aseptic manufacturing, gamma/e-beam adaptations), cryopreservation of cell-laden precursors and validated loading of biologics (antibodies, growth factors, viral vectors) within injectable natural hydrogels have addressed early barriers to regulatory approval [40,59]. These process innovations are critical for first-in-human trials of drug- or cell-carrying gels, and (iii) local immunomodulation and combination therapies: injectable hydrogels are increasingly engineered to act as local immuno-modulatory depots, releasing cytokines, checkpoint inhibitors or stimulator of interferon genes agonists in a controlled fashion to synergize with systemic therapies. Recent preclinical studies show that perioperative or intratumoural in situ hydrogels can enhance local anti-tumour immunity and reduce systemic toxicity, and several translational programs are preparing clinical translation in oncology and postoperative settings [154].

In summary, biocompatible, stimuli-sensitive natural hydrogels offer a compelling suite of functionalities across drug delivery, tissue engineering, wound healing, biosensing and injectable therapies (Table 7). Their synergy of dynamic responsiveness and biological compatibility positions them as key materials for next-generation biomedical platforms. Yet widespread translation requires overcoming limitations in mechanical robustness, reproducibility, scalability, and reliable in vivo performance.

The following chapter will examine emerging trends, to explore how current research is addressing the existing challenges and what the future may hold for these intelligent biomaterials.

## 4. Emerging Trends

The past decade has witnessed rapid progress in the design, characterization, and clinical translation of biocompatible stimuli-sensitive natural hydrogels, largely driven by multidisciplinary advances in polymer chemistry, nanotechnology, and biofabrication. These developments have shifted the field from proof-of-concept laboratory studies to first-in-human trials exploring injectable, adaptive, and personalized hydrogel therapies. The emerging technological innovations in hydrogel engineering directly build upon and strategically address the practical limitations identified across current biomedical uses, pointing toward increasingly intelligent and integrated material platforms. Current progress can be grouped into five major fronts: hybrid/nanocomposite systems, self-healing and shape-memory matrices, 3D bioprinting and microfabrication, personalized and precision medicine applications, and AI-guided or computationally modeled hydrogel design.

### 4.1. Hybrid and Nanocomposite Hydrogel Systems

Recent years have witnessed significant progress in developing hybrid and nanocomposite hydrogels that integrate natural polymers with synthetic or nanoscale components to enhance mechanical integrity, responsiveness, and functionality. The rationale behind hybridization is to overcome the inherent limitations of purely natural matrices, such as weak mechanical strength or rapid degradation, while preserving biocompatibility and biofunctionality [163]. Hybridization and nanocomposite design strategies have markedly expanded the mechanical strength, conductivity, and multifunctionality of natural hydrogels [164,165]. Incorporation of nanoparticles, nanoclays, or nanofibers into natural polymer networks has yielded stimuli-responsive nanocomposites capable of precise drug release and enhanced cellular interactions [20,166]. Nanomaterials such as graphene oxide, silica nanoparticles, and metal–organic frameworks [167,168] have been incorporated to impart electrical responsiveness, photothermal capability [169], or enhanced load bearing while retaining the hydrogel’s intrinsic biocompatibility. For example, although hydrogel is the most widely utilized biomaterial for cardiac tissue engineering, it has some limitations, including a lack of electrical conductivity and appropriate mechanical properties, which are crucial in cardiac function. Chitosan-graphene oxide composites exhibited superior mechanical reinforcement and superior conductivity, supporting neuronal cell adhesion and controlled release of neurotrophic factors, promoting neuronal differentiation in vivo in spinal cord injury models [170]. Similarly, alginate-silica nanocomposites have been developed for injectable bone and cartilage regeneration, where the silica phase proves osteoinductive and improves crosslinking stability and compressive properties, supporting bone regeneration in rabbit critical-sized defects [171]. A particularly promising direction is nanoparticle-mediated responsiveness: metallic (Au, Fe_3_O_4_) and polymeric nanoparticles incorporated within natural matrices confer remote control through magnetic, optical, or electrical stimuli. For example, magnetically responsive gelatin-Fe_3_O_4_ hydrogels have demonstrated spatially controlled drug release under alternating magnetic fields [172,173]. Likewise, HA-gold nanocomposites have enabled localized photothermal modulation for tumor microenvironment regulation [174,175]. Hybridization has also improved cell-instructive behavior, representing an important bridge between soft biomaterials and structurally demanding tissues. Silk fibroin-HA hybrids composites, for instance, have shown synergistic mechanical resilience and bioactivity, guiding stem cell differentiation in 3D culture systems, facilitating ECM deposition by mesenchymal stem cells [44,176]. These multifunctional hybrids highlight a shift from single-function matrices toward multimodal platforms capable of responding to diverse physiological environments and supporting complex tissue repair, a growing emphasis being placed on functionally graded designs that combine the biological familiarity of natural polymers with the tunability of engineered nanophases.

### 4.2. Self-Healing and Shape-Memory Hydrogels

Another emerging trend is the engineering of self-healing and shape-memory natural hydrogels, mimicking the adaptability of living tissues. These materials possess reversible bonding motifs, such as hydrogen bonds, Schiff bases, boronate esters, or host-guest interactions, that allow structural recovery after mechanical damage [163,177,178,179,180]. Such responsiveness enhances injectability, extends service life, and enables minimally invasive deployment in irregular defects. Chitosan-based dynamic hydrogels crosslinked via imine or boronate ester bonds exploit reversible diol-boronate bonds, producing rapid (<1 min) self-repair while maintaining injectability and antibacterial activity, ideal for wound repair and mucosal sealing [181]. In tissue engineering, alginate-gelatin hybrid hydrogels with reversible ionic and hydrogen bonds restore structural integrity post-deformation, maintaining 3D cell viability in cyclic load environments [182].

Shape-memory behavior, in contrast, enables hydrogels to recover their programmed shape upon exposure to specific stimuli such as temperature or pH changes. Silk fibroin- poly(N-isopropylacrylamide) composites, for instance, exhibit thermally triggered contraction that can aid in post-surgical wound closure [183,184,185,186]. Furthermore, oxidized alginate-chitosan imine networks show reversible imine exchange, allowing the hydrogels to recover their shape by pH variation, and seal bone defects after deformation, offering potential in minimally invasive implant delivery [187,188].

The combination of reversible chemistry with biocompatible scaffolds not only simplifies surgical handling but also aligns with regulatory expectations for biodegradable, mechanically stable implants. Self-healing underscores a broader trend toward adaptive biomaterials capable of repairing themselves and responding to mechanical stress in vivo. These dynamic systems have entered translational evaluation: preclinical animal models have validated self-healing chitosan hydrogels for dural sealing and nerve conduit repair [180,189], while early feasibility studies are exploring shape-memory HA hydrogels as conformal cartilage fillers [190]. Dynamic HA-based self-healing hydrogels are under evaluation for conformal cartilage repair with preliminary data indicating excellent biocompatibility and viscoelastic recovery [191,192].

### 4.3. 3D Bioprinting and Microfabrication of Smart Hydrogels

The convergence of 3D bioprinting and stimuli-responsive natural hydrogels has created precision-engineered constructs capable of reproducing native tissue gradients and architectures, revolutionizing biofabrication strategies for regenerative medicine [193,194,195]. Advances in dual crosslinking strategies, such as photopolymerization coupled with ionic or enzymatic gelation, have enabled spatial control over stiffness and degradation rates [196]. Natural polymers such as alginate, gelatin-methacrylate, chitosan, and HA have been engineered into printable bioinks with tunable rheology, crosslinking kinetics, and cell-supportive microenvironments. Recent advances focus on multi-material printing and in situ crosslinking, enabling complex architectures with spatially defined mechanical and biochemical properties [197,198]. The creation and/or modification of interpenetrating interconnecting networks, reinforcement of layer-layer interactions, and modification of ink formulations enhance the structural integrity of gelatin-methacrylate bioprinting scaffolds [199]. Enzyme-triggered (using horseradish peroxidase-H_2_O_2_ systems) chitosan bioink hydrogels have been used in extrusion bioprinting for constructing tissue scaffolds under mild conditions compatible with living cells [200].

The advent of 4D bioprinting-where time-dependent environmental triggers induce shape or function changes-has further expanded design possibilities. Thermo- and pH-sensitive HA-based bioinks now allow post-print morphing for dynamic tissue models [201]. Integration of microfabrication and microfluidic technologies has also enabled biosensor-laden hydrogels with embedded microchannels for continuous monitoring of metabolites or oxygen tension [202].

Translationally, bioprinted cartilage and skin constructs using natural hydrogels are being clinically evaluated, marking one of the first cell-laden natural bioink systems entering human testing [203]. Together, these results confirm the growing feasibility of patient-specific tissue constructs derived from stimuli-sensitive, cell-compatible hydrogels. As biofabrication matures, combining precision architecture with biological adaptivity positions natural stimuli-responsive hydrogels at the forefront of regenerative engineering.

### 4.4. Personalized and Precision Medicine Applications

Stimuli-sensitive hydrogels are increasingly aligned with precision medicine, offering patient-specific therapeutics, diagnostics, and regenerative approaches, serving as personalized delivery depots that can adapt drug release kinetics to individual pharmacokinetic profiles or local tissue microenvironments. Advances in bioprinting, CM, and biopolymer chemistry now enable custom-designed hydrogel formulations tailored to patient physiology and disease context [204,205]. In regenerative medicine, 3D bioprinting of autologous-cell-laden hydrogels (e.g., HA or collagen matrices) enables individualized tissue constructs guided by patient imaging data [206]. Pilot human trials for patient-specific cartilage and skin constructs (NCT06897098 [207]) demonstrate the feasibility of combining patient-derived cells with responsive scaffolds for precision healing outcomes. Examples include enzyme-triggered depots for postoperative chemotherapy, immunomodulatory gels [208], and closed-loop metabolic control systems: pH- and enzyme-responsive HA-peptide hybrid hydrogels have been explored for patient-tailored release of anticancer biologics in resected tumor beds, representing a prototype for individualized local therapy [209]. Likewise, chitosan-based depot systems personalized for intratumoral immunotherapy, matching local enzymatic degradation rates, have demonstrated localized checkpoint-inhibitor delivery with sustained immune activation [210].

An emerging subfield integrates biosensing and therapeutic response within a single hydrogel platform—“theranostic hydrogels” For example, in metabolic control, glucose-responsive chitosan hydrogels capable of feedback-controlled insulin release have been validated in small feasibility studies [211,212,213]. These approaches exemplify the transition from “smart materials” toward “theranostic” and patient-specific hydrogel biointerfaces, capable of self-adapting to dynamic physiological conditions, merging sensing, actuation, and therapy.

### 4.5. Artificial Intelligence and CM in Hydrogel Design

Artificial intelligence (AI) and CM are emerging as transformative tools in rational hydrogel design and optimization. ML algorithms have been trained on large experimental datasets to forecast gelation behavior, mechanical modulus, and drug-release kinetics for natural polymer combination datasets [214]. Computer simulations now enable quantitative predictions of in vivo swelling, diffusion, and degradation profiles, assisting both design optimization and regulatory submissions [215]. Computational screening can identify optimal crosslinking densities or stimulus-responsive motifs for desired degradation or release profiles. For instance, data-driven modeling can been used to optimize pH-responsive chitosan-alginate hydrogels for oral peptide delivery [216]. Meanwhile, finite element modeling allows simulation of in vivo deformation and diffusion, aiding in the design of injectable hydrogels with predictable spatial gelation [217].

AI integration also accelerates clinical translation: predictive analytics help correlate preclinical metrics (mechanical strength, degradation, cytocompatibility) with human trial outcomes, streamlining regulatory approval. Furthermore, AI-driven robotic synthesis platforms can automatically generate and test hydrogel formulations, optimizing patient-specific compositions for therapeutic applications [218]. These advances could enable on-demand synthesis of patient-tailored hydrogels in hospital settings. Integration of computational and experimental workflows is expected to shorten development cycles and enhance reproducibility, long-standing challenges in the hydrogel field, while paving the way for data-rich, regulatory-ready material platforms. Such computationally guided pipelines will likely define the next generation of smart, adaptive biomaterials, embedding predictive intelligence into hydrogel design. Collectively, all those advances underscore a paradigm shift: stimuli-sensitive natural hydrogels are evolving from laboratory curiosities to clinically validated adaptive biomaterials. Hybridization, dynamic crosslinking, and data-driven optimization have improved their tunability and reproducibility, while early human trials confirm real translational potential. The next phase will likely integrate multi-stimuli responsiveness, biofabrication, and AI-assisted formulation within the same framework-bringing the promise of personalized, intelligent biomaterials closer to clinical reality.

The convergence of materials chemistry, nanotechnology, AI, and biofabrication is propelling natural stimuli-sensitive hydrogels from laboratory research into clinical reality. The field is transitioning from proof-of-concept prototypes toward clinically validated, multifunctional systems capable of sensing, responding, and healing. Nonetheless, challenges remain in scaling reproducible manufacturing, ensuring regulatory compliance, and achieving long-term biostability. Future research should emphasize mechanistic understanding of in vivo stimuli response, integration with biosensors and drug reservoirs, and standardized evaluation protocols bridging preclinical and human data. Ultimately, hybridized, adaptive, and AI-informed hydrogel technologies will anchor a new generation of intelligent biomaterials for regenerative and personalized medicine.

## 5. Challenges and Limitations

Despite remarkable progress in the design and biomedical translation of stimuli-sensitive natural hydrogels, several challenges continue to impede their clinical translation: mechanical performance and in vivo stability, reproducibility and GMP-scale manufacture, regulatory uncertainty for combination/theranostic products, and ethical/sustainability issues that next-generation technologies must strategically overcome. Below, we summarise these limitations, illustrate why they matter for translation, and point to mitigation strategies.

### 5.1. Mechanical Strength and Structural Stability

Natural hydrogels are inherently hydrated, low-modulus networks, exhibiting low mechanical strength and rapid degradation under physiological stress. Their limited toughness, brittleness under cyclic loading and trade-offs between stiffness and permeability constrain use in load-bearing applications and in dynamic tissue environments, such as cartilage, tendon, and myocardium [219]. Mitigation strategies include the design of dual-crosslinked networks (fast physical gelation + slower covalent reinforcement), incorporation of bioinert reinforcing phases with demonstrated long-term biocompatibility, and standardized mechanical testing protocols (static, dynamic fatigue, and swelling-stiffness mapping) to correlate in vitro metrics with in vivo outcomes [8,220,221]. Double-network, interpenetrating networks and nanocomposite reinforcement (e.g., cellulose nanocrystals, silica, graphene derivatives) have demonstrably improved toughness and fatigue resistance in preclinical models, but implementation often introduces new issues (e.g., altered biodegradation, potential cytotoxicity of nanofillers) that must be addressed case-by-case [8]. While these hybrid methods have improved modulus and toughness, they often compromise the hydrogel’s injectability or biodegradability. Moreover, long-term fatigue testing and mechanical validation in dynamic environments remain insufficiently standardized. For example, reproducible mechanical benchmarks for injectable chitosan-β-glycerophosphate thermogels in cartilage applications are still lacking [16]. Moreover, standard mechanical testing under physiologically relevant cyclic and multiaxial loading is not yet harmonized across the field, making cross-study comparisons difficult. An additional mechanical concern arises from in vivo degradation-stiffness mismatch. Hydrogels designed to degrade synchronously with tissue remodeling frequently lose mechanical integrity before sufficient regeneration occurs, particularly in ischemic or enzymatically active sites [221]. This issue underscores the importance of adaptive crosslinking and multi-stimuli responsive designs capable of real-time mechanical adjustment to tissue evolution.

### 5.2. Reproducibility and Scalability Challenges

Reproducibility across research laboratories and scalability to clinical-grade manufacturing remain among the most pressing barriers. Natural polymers vary by source and batch, small variations in polymer source, for instance, chitosan sourced from different crustaceans or alginate from different seaweed species, can differ in degree of acetylation (for chitosan), or mannuronate-guluronate ratios. Deacetylation or molecular weight distribution (for HA and alginate) significantly alter gelation kinetics, producing substantial lot-to-lot differences in viscosity and mechanical properties [222]. Such variability complicates formulation transfer from academia to GMP manufacture and undermines reproducibility. Moreover, sterilization (gamma, ethylene oxide, aseptic fill) and endotoxin control can modify labile functional groups used for stimuli-responsiveness, requiring validated, material-specific sterilization routes [223]. These manufacturing and process-control gaps are a frequent bottleneck for IND/IDE filings.

Another scalability limitation is the energy and cost intensity of purification. Endotoxin removal, sterilization, and lyophilization steps are complex and prone to altering polymer chemistry. HA and collagen are particularly sensitive to sterilization methods, often requiring low-temperature or aseptic in situ crosslinking [224]. These constraints complicate clinical translation, particularly for multi-component hybrid systems. Industrial-scale hydrogel production must adopt well-characterized raw material specifications, implement orthogonal analytical QC (molecular weight, endotoxin, free-functional group assays), and use process analytics and design-of-experiments together with pilot Good Manufacturing Practice runs to establish robust scale-up windows, in order to meet ISO 13485 standards [225]. Digital manufacturing and AI-driven quality control pipelines can enhance reproducibility. ML tools can predict rheological and degradation properties based on raw material characteristics, and AI/ML-assisted predictive QC is emerging as a useful tool to flag out-of-spec batches early [226], but integration into regulatory workflows remains early-stage.

### 5.3. Regulatory and Translational Barriers

The regulatory landscape for stimuli-sensitive natural hydrogels is still evolving. Many formulations straddle multiple categories, medical device, drug-device combination, or biologic-depending on their intended use, payload, and degradation profile. This ambiguity (device vs. drug vs. combination product) complicates classification under regulatory frameworks such as the U.S. Food and Drug Administration, European Medicines Agency, and China National Medical Products Administration. Sponsors must navigate combination-product guidance and device-drug interface expectations (e.g., human factors, biocompatibility, long-term degradation products) [227,228]. Regulators increasingly require extended biodegradation, immunotoxicology, and leachables profiling for degradable responsive networks-data packages that are expensive and time-consuming to generate. Additionally, lack of harmonized standards for “smart” responsive materials complicates regulatory review. For instance, HA-based in situ gelling systems for cartilage repair (e.g., Hy2Care CartRevive^®^) are reviewed under device-biologic combination regulations (NCT05186935 [80]), whereas enzyme-responsive chitosan depots delivering immunomodulators [229] fall under drug-device designations. The absence of a harmonized international standard for smart or stimuli-sensitive biomaterials results in inconsistent evaluation metrics for biocompatibility, immunogenicity, and degradation by-products [230].

Furthermore, long-term biosafety data-including metabolite clearance, inflammatory responses, and systemic toxicity-are limited. Regulatory agencies increasingly demand multi-month degradation tracking and immunotoxicology profiling, yet these requirements are difficult to fulfill for biodegradable materials that degrade differently across tissues and patients. Another regulatory concern involves intellectual property complexity: many stimuli-sensitive formulations combine proprietary polymers, nanoparticles, and bioactive agents. Ensuring freedom-to-operate (FTO) and patent clarity remains a hurdle to commercial adoption.

Mitigation strategies include early regulatory engagement, clarify primary mode of action to determine lead regulatory center, and plan integrated device-drug toxicology and biocompatibility testing with ISO 10993-series endpoints tailored for degradable materials [231]. Collaborative consortia to define standards for stimuli testing (e.g., enzyme levels, redox potentials used as release triggers) would accelerate consistent evaluation.

### 5.4. Ethical, Environmental, and Sustainability Considerations

While natural hydrogels are often regarded as “green” materials, their large-scale production raises ethical and environmental concerns. Chitosan extraction depends heavily on crustacean shell waste, which varies seasonally and regionally. Similarly, animal-derived collagen and gelatin raise issues related to source traceability, zoonotic safety, and religious/ethical constraints [232]. Synthetic analogues or recombinant production systems are being explored but remain expensive and energy-intensive. Downstream, the environmental footprint of purification, sterilization and cold-chain logistics can be substantial if not optimized [233].

Biodegradability, though advantageous biologically, can create challenges in storage stability and transport. Formulations with labile bonds (e.g., imine, disulfide) may degrade under humidity or temperature stress during shipping, necessitating cold-chain logistics that offset environmental gains. Furthermore, hydrogel remnants and polymeric waste streams from clinical production are rarely assessed for ecotoxicity-an emerging priority under EU REACH [234] and ISO 10993-1 amendments [231]. End-of-life and environmental fate of degraded polymer fragments (especially with embedded nanomaterials) is under-studied.

Ethically, personalized hydrogel systems that incorporate patient-derived cells or AI-driven composition adjustment raise questions about data ownership, algorithmic transparency, reproducibility of patient-specific outcomes, and equitable access. Ensuring that such smart biomaterials remain equitable, affordable, and environmentally sustainable will be central to their long-term societal acceptance.

Overcoming these challenges requires coordinated efforts between material scientists, clinicians, and regulatory bodies. Transition toward renewable feedstocks (plant-derived polysaccharides, recombinant proteins), greener chemistries (aqueous, enzyme-mediated crosslinking) and lifecycle analyses early in development will improve sustainability, while harmonized testing standards, early regulator engagement, and harmonized regulatory guidelines will be key to ensuring safety and reproducibility. Coupling standardized experimental benchmarks with computational, AI-assisted design and predictive quality control will accelerate reproducible, safe and scalable hydrogel platforms ready for broader clinical adoption. The convergence of hybrid design, data-driven modeling, and sustainable manufacturing is poised to transform stimuli-sensitive natural hydrogels into clinically robust, ethically sound, and environmentally responsible biomaterials. Table 8 correlates the main challenges described with emerging trends in natural stimuli-responsive hydrogels.

## 6. Future Perspectives

**Figure 12:**
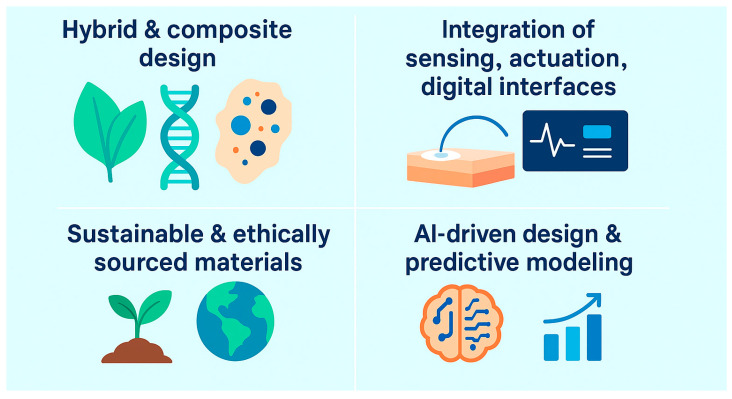
Prospective advances for next-generation stimuli-sensitive hydrogels.

Stimuli-sensitive natural hydrogels have matured significantly, the next generation promises to transcend current limitations through intelligent design, integration with digital and manufacturing technologies, and a stronger commitment to clinical translation and sustainability. While the past decade has focused on fundamental mechanisms and preclinical feasibility, the immediate future is likely to be defined by four convergent advances: the design and integration of multi-responsive hybrid systems; advances in biofabrication and smart material design; exploitation of AI/CM; and the embedding of sustainability into clinical translation (Figure 12). Together, they represent a pathway from current proof-of-concepts toward robust, patient-specific therapeutic platforms. Below we provide an outlook.

### 6.1. Integration of Multi-Responsive and Hybrid Systems

Single-stimulus hydrogels (e.g., pH or thermal) have demonstrated targeted drug release and regenerative performance [51], yet clinical environments are far more complex. Tissues and pathological microenvironments often present multiple overlapping triggers, for example, pH shifts, redox gradients, enzymatic activity, mechanical stress and external triggers like light or magnetics. To meet this complexity, hybrid hydrogels that combine two or more responsive motifs (e.g., pH + redox, thermo + enzyme) or that incorporate nanophases (magnetic, photothermal) are increasingly engineered to respond to two or more stimuli in concert, allowing richer, context-dependent responses. Recent progress [163] has focused on multi-responsive hybrid hydrogels, which combine natural biopolymers (chitosan, alginate, collagen, HA) with inorganic or synthetic components (e.g., polypyrrole, graphene oxide, gold nanoparticles) to introduce dual or triple sensitivity. For example, injectable “smart” hydrogels that respond to pH, temperature and magnetic fields simultaneously and are built from natural/synthetic composites [235]. Alginate-graphene composites exhibited synchronized pH/redox-driven degradation and were validated for on-demand delivery of doxorubicin in breast cancer xenografts [236]. Dual-responsive alginate/graphene or chitosan-enzyme systems enable triggered release only under specific disease microenvironments, reducing off-target payload leakage and improving therapeutic indices in animal models [237,238]. These materials can integrate pH and redox dual-responsiveness, enabling precise control over drug release in inflamed or cancerous microenvironments. Such designs open opportunities for trigger-specific, context-aware release, reduced off-target interactions, and better adaptability to dynamic tissue environments. Similarly, a thermo-enzyme dual-responsive chitosan-gelatin hydrogel accelerated osteogenic differentiation under local enzymatic activity in a rat calvarial defect model [239]. Continued focus will be on minimizing toxicology from added nanophases and guaranteeing predictable degradation [8,20]. The convergence of bioelectronic responsiveness is an emerging direction: electrically and magnetically sensitive hydrogel scaffolds allow external modulation of cell migration, angiogenesis, and localized release [240,241]. Clinical translation will depend on minimizing toxicity from conductive fillers and optimizing remote activation protocols.

The road ahead will require ensuring that added responsiveness does not compromise safety, degradability, or biocompatibility, particularly where nanomaterials are incorporated. Ultimately, multi-responsive systems must evolve toward self-adaptive biomaterials capable of sensing and responding autonomously to physiological feedback, akin to a biological reflex. Progress in this direction will rely heavily on materials, informatics and real-time biosensing integration.

### 6.2. Advances in Biofabrication and Smart Material Design

Progress in 3D bioprinting, microfluidics, and in situ crosslinking are revolutionizing the capacity to spatially pattern responsiveness, architecture and mechanical properties of stimuli-sensitive hydrogels. In situ crosslinking enables spatial patterning of responsiveness and mechanical gradients that better mimic native tissue architecture. Advances in extrusion-based and volumetric printing now permit fabrication of hydrogel constructs with micron-scale precision, embedding cells, growth factors, and multiple responsive domains simultaneously. Recent developments include dual-crosslink bioinks (e.g., light-triggered and enzyme-mediated crosslinking) enable spatial patterning of responsiveness within a single construct [242] with secondary maturation crosslinks for long-term stability, while volumetric and extrusion methods are achieving cell-compatible speeds and resolutions suitable for clinical translation [89,243,244]. A recent review outline how dual-crosslinking strategies (e.g., photopolymer + ionic/enzymatic) allow printed constructs to have immediate shape fidelity and long-term stability [245]. In situ gelation injectables are also evolving into minimally invasive delivery vehicles that crosslink on site and adapt to tissue defects [233]. Importantly, early clinical and feasibility efforts are now exploring printed, cell-laden constructs for cartilage and soft-tissue repair, signaling a shift toward patient-specific regenerative implants [59,246]. 3D bioprinted hybrid collagen-HA hydrogel scaffold exhibit regional pH and temperature sensitivity for osteochondral interface regeneration [247]. Such complex architectures are essential for recapitulating natural tissue heterogeneity and gradient-based morphogenesis. Future applications will benefit from: (i) multi-material printing combining responsive and inert domains; (ii) embedded sensing/actuator elements for real-time feedback; (iii) integration with cell-laden biomaterials for personalised regenerative implants. The future likely lies in modular manufacturing platforms, where hydrogel building blocks with defined stimuli responsiveness are assembled on demand for specific patient or disease contexts. A key challenge remains matching the printing kinetics, cell compatibility, and stimuli responsiveness while retaining scalability and sterilisation compatibility.

### 6.3. AI and CM in Hydrogel Design

The integration of AI and CM into hydrogel design has expanded rapidly. As formulation variables expand (polymer types, crosslinkers, stimuli-sensitivity, nanofillers), AI, ML and CM simulations are becoming essential tools to optimise hydrogel design. Recent studies show ML models predicting printability, mechanical properties and drug-release kinetics from polymer composition data [248]. Meanwhile, finite element modelling and digital twin frameworks now model hydrogel-tissue interactions and swelling/degradation behaviour under physiological conditions [40]. Predictive models based on finite element analysis, molecular dynamics, and ML are now capable of simulating network swelling, drug diffusion, and degradation kinetics across complex physiological environments [202]. Beyond structure, computational design and AI-driven optimization are transforming hydrogel engineering. ML models trained on rheological and degradation datasets can predict composition-property relationships and suggest novel polymer blends with target mechanical or kinetic profiles [249]. Coupled with high-throughput robotic synthesis, this approach reduces experimental trial-and-error and accelerates regulatory documentation.

AI, ML and CM accelerate hydrogel discovery and optimization by mapping composition-properties and predicting gelation/degradation behavior. Recent reviews summarize how ML models predict printability, gel modulus, and drug-release kinetics from formulation descriptors; experimental demonstrations show ML-guided optimization reduces experimental rounds substantially [225,250]. Integration of finite-element-based digital twins with patient imaging will enable personalized hydrogel constructs with pre-validated in vivo performance. Data standards and open datasets remain critical to scale these approaches. AI-driven inverse design frameworks can specify polymer composition or crosslink density required to achieve desired responsiveness thresholds. For instance, a generative ML model can identify new polysaccharide-peptide hybrid gels with improved mechanical resilience and targeted degradation profiles [25]. Additionally, digital twin models are being developed to simulate hydrogel-tissue interactions, incorporating patient-specific anatomy, tissue stiffness, and inflammatory biomarkers. This concept is being tested to match computational predictions and clinical pharmacokinetics [251].

These tools can drastically reduce trial-and-error development cycles, improve reproducibility, and support regulatory dossiers. For the next stage, accessible hydrogel performance databases and standardised high-throughput experimental-data linkages will be critical to realise AI-driven material discovery. However, the data standardization challenge persists datasets describing hydrogel rheology, degradation, and biological performance remain fragmented. Establishing open-access hydrogel databases, similar to protein or materials genome initiatives, will be essential for AI reliability and reproducibility.

### 6.4. Toward Sustainable and Clinically Translatable Hydrogel Technologies

Sustainability and clinical readiness must be embedded into early-stage hydrogel design from the outset, rather than considered as add-ons. Natural polymers offer ecological and safety advantages but often require energy-intensive processing, variable sourcing and cold-chain logistics. Recent analyses emphasise the need for sourcing polysaccharides from renewable feedstocks and agro-waste streams, green crosslinking chemistries adopting enzymatic (solvent-free) functionalisation, life-cycle assessments, selecting sterilization/storage approaches compatible with labile stimuli chemistries, and end-of-life disposal strategies [252,253,254,255,256]. Life-cycle analyses and early dialogue with regulators regarding combination-product classification will accelerate translation and reduce late-stage redesign. Circular bioeconomy models are now being explored, where marine- or plant-derived polysaccharides (e.g., alginate, cellulose, dextran) are sourced from renewable or waste biomass. Life cycle analyses suggest that replacing synthetic additives with plant-based alternatives can reduce environmental impact during production.

Moreover, bioelectronics-hydrogel integration offers a route toward continuous monitoring and adaptive therapy. For instance, implantable hydrogel-sensor arrays for monitoring glucose and pH in surgical wound beds under clinical evaluation [257]. Such systems could soon enable feedback-controlled release of therapeutics directly from the hydrogel matrix [126].

Clinically, the next decade will likely witness an expansion of personalized hydrogel systems, guided by AI-assisted formulation and point-of-care 3D printing. The growing regulatory experience from HA cartilage and chitosan wound platforms will provide a foundation for accelerated pathways of multi-responsive systems.

Regulatory pathways for dynamic, stimuli-responsive hydrogels (especially combination device-drug products) remain complex. Early engagement with regulators, standardised test-beds and validated manufacturing pathways will accelerate translation and reduce late-stage attrition. The long-term vision is for digitally designed, sustainably produced, patient-specific hydrogels that function as real-time therapeutic platforms. Finally, cross-disciplinary collaboration-uniting materials science, AI, regulatory policy, and clinical practice-will determine the translational success of the field. The long-term vision is to establish digitally designed, sustainably produced, patient-specific hydrogel systems that function as dynamic biointerfaces, advancing from passive scaffolds to active, intelligent therapeutic platforms

Looking forward, the field of stimuli-sensitive natural hydrogels is poised to evolve from passive scaffolds to active, adaptive, clinically robust biomaterials that combine sensing, actuation and therapy. The convergence of hybrid chemistry, precision biofabrication, AI-driven optimisation and sustainable manufacturing will define the next decade of innovation.

Realising this vision will require multidisciplinary coordination: materials scientists, bioengineers, computational scientists, clinicians and regulatory bodies must work together to translate promising platforms from bench to bedside. Key enablers will be harmonized testing standards, open data infrastructures to feed ML pipelines, greener processing chemistries, and regulatory frameworks that accommodate dynamic, combination-product biomaterials.

## 7. Conclusions

Stimuli-sensitive natural hydrogels have evolved from simple water-swollen polymer networks to multifunctional, clinically relevant biomaterials capable of responding dynamically to the physiological media. Their ability to translate biocompatibility, biodegradability, and bioactivity into programmable therapeutic functions continues to drive innovation in drug delivery, tissue engineering, wound care, and biosensing. The past decade has witnessed a decisive shift from proof-of-concept laboratory systems to translational and early clinical studies, as exemplified by injectable alginate cardiac scaffolds and HA-based cartilage fillers progressing through regulatory approval. These milestones signal that stimuli-sensitive natural hydrogels are moving steadily from bench to bedside.

A key conclusion emerging from this review is that hybrid and composite design strategies are central to overcoming the intrinsic weaknesses of natural polymers, particularly their mechanical fragility and limited tunability. Reinforcing bio-based matrices with nanoscale fillers or dynamic crosslinking motifs has resulted in constructs that retain biocompatibility while gaining robustness and controlled responsiveness. These advances open routes toward personalized medicine, where hydrogel formulations can be tailored to patient-specific biomechanics, degradation kinetics, and cellular environments.

Equally transformative is the integration of sensing, actuation, and digital interfaces. Smart hydrogel dressings and microneedle sensors now demonstrate closed-loop therapeutic and diagnostic capabilities. Such systems exemplify the convergence of biomaterials with bioelectronics, creating real-time feedback networks that could redefine postoperative monitoring, wound management, and chronic disease care. The convergence of hydrogel science with AI-driven design and predictive modeling is expected to accelerate optimization, enabling the rational selection of polymer-crosslink combinations for desired mechanical, transport, and biological outcomes.

However, several challenges remain before full clinical translation is realized. Reproducibility in natural polymer sourcing, batch-to-batch consistency, and sterilization methods continue to complicate regulatory approval. Long-term biocompatibility and controlled degradation must be demonstrated through standardized in vivo and GLP-compliant studies. Moreover, ensuring scalable manufacturing without compromising structural fidelity is critical for industrial deployment. Addressing these constraints will require harmonized frameworks linking material chemistry, process engineering, and regulatory science.

Looking forward, future research should prioritize sustainable and ethically sourced biomaterials, integration of multi-responsive hybrid systems, and coupling of biofabrication with data-guided personalization. The vision is clear: a new generation of intelligent, bioinspired hydrogel technologies that actively participate in healing and monitoring rather than passively supporting tissues. Achieving this vision will depend on interdisciplinary collaboration among polymer chemists, clinicians, and data scientists-transforming stimuli-responsive natural hydrogels into the cornerstone of next-generation regenerative and precision medicine.

## Figures and Tables

**Table 1:** Summary of natural polymers in stimuli-responsive hydrogels for biomedical applications.

Natural Polymer	Primary Stimuli- Responsiveness	Crosslinking Type	Key Biomedical Applications	Main Challenges
Chitosan	pH, temperature	Physical/Chemical	Drug delivery, wound healing	Weak mechanics, rapid degradation
Alginate	pH, ionic strength	Ionic/Physical	Encapsulation, tissue scaffolds	Poor cell adhesion
Collagen/ Gelatin	Enzymatic, temperature	Chemical/Physical	Tissue engineering	Fast degradation, low stiffness
HA	Enzymatic, redox	Covalent	Regenerative medicine, drug release	Mechanical reinforcement
Cellulose/Dextran	pH, enzyme, redox	Chemical	Controlled release, biosensing	Complex modification
Silk fibroin	pH, enzyme	Physical/Chemical	Tissue repair, implants	Limited solubility, processing issues

**Table 2:** Types of Stimuli and Corresponding Hydrogel Responses.

Stimulus	Responsive Moiety/Mechanism	Example Polymers	Typical Response	Biomedical Application
pH	Protonation/deprotonation of ionizable groups	Chitosan, Alginate	Swelling/deswelling	Oral drug delivery, tumor targeting
Temperature	LCST/UCST transitions	Gelatin, Chitosan/β-GP	Sol–gel transition	Injectable scaffolds
Enzyme	Enzymatic cleavage of linkers	Collagen, HA, Gelatin	Degradation, drug release	Tissue regeneration
Redox	Disulfide bond cleavage	HA, Dextran	Gel degradation, drug release	Cancer therapy
Ionic strength	Ion exchange/crosslinking	Alginate, Carrageenan	Swelling control	Cell encapsulation
Multi-stimuli	Synergistic triggers	Chitosan-Gelatin, HA hybrids	Complex adaptive response	Precision medicine

**Table 3:** Structural and Chemical Characteristics of Natural Polymers Used in Stimuli-Sensitive Hydrogels.

Polymer	Main Functional Groups	Natural Source	Crosslinking Modes	Degradation Mechanism	Biological Advantages
Chitosan	-NH_2_, -OH	Crustacean shells	Ionic, covalent	Lysozyme degradation	Antimicrobial, mucoadhesive
Alginate	-COOH	Brown algae	Ionic (Ca^2+^), covalent	Hydrolysis	Mild gelation, cell encapsulation
Collagen/Gelatin	-NH_2_, -COOH, peptide chains	Animal connective tissue	Enzymatic, covalent	MMP-mediated	Cell adhesion, ECM mimicry
HA	-COOH, -OH	Animal tissues, bacteria	Covalent, enzymatic	Hyaluronidase degradation	ECM mimicry, angiogenic
Silk fibroin	-NH_2_, -COOH	Silkworm cocoon	Physical (β-sheet), covalent	Protease degradation	High tensile strength
Dextran	-OH	Microbial polysaccharide	Covalent	Hydrolysis	Biocompatible, modifiable
Cellulose	-OH	Plant biomass	Covalent, physical	Hydrolysis	Reinforcement, mechanical strength

**Table 4:** Stimuli-Sensitive Natural Hydrogel Systems for Controlled Drug Delivery.

Stimulus Type	Representative Natural Hydrogel System	Typical Payloads	Release Mechanism	Biomedical/Clinical Application	Refs.
pH- responsive	Chitosan/alginate composite; carboxymethyl cellulose	Small- molecule drugs (DOX, 5-FU), peptides	Protonation/deprotonation alters swelling and ionic interactions → diffusion-controlled release	Targeted anticancer therapy in acidic tumour microenvironment; oral delivery through intestinal pH gradients	[11,27]
Redox- responsive	Hyaluronic acid or gelatin hydrogels with disulfide or thioketal linkages	Antibodies, siRNA, cytokines	Reduction of disulfide bonds by intracellular GSH → cleavage and drug release	Tumour microenvironment-specific release; local antibody delivery	[69,76]
Enzyme- responsive	Collagen/gelatin or chitosan hydrogels with MMP-cleavable peptides	Growth factors, ECM proteins, antibiotics	Local enzymatic degradation by MMPs, lysozyme or collagenase	Wound healing, tissue regeneration, anti-fibrotic therapy	[26,78]
Thermo- responsive	Chitosan/β-glycerophosphate; gelatin; methylcellulose blends	Proteins, cells, small-molecule drugs	Sol–gel transition at physiological temperature → depot formation	Injectable in situ forming gels for localized chemotherapy, pain relief, and cartilage repair	[28,79]
Electrolyte/Ionic- responsive	Alginate-Ca^2+^ or κ-carrageenan systems	Peptides, antibiotics, vitamins	Ion exchange or chelation alters crosslink density and swelling	Oral and mucosal delivery; sustained nutrient or antimicrobial release	[59,75]
Light- responsive (secondary hybrid)	HA-silk fibroin with photolabile linkers or embedded gold nanoparticles	Anticancer agents, photothermal drugs	Light-induced bond cleavage or thermal softening → burst release	On-demand local tumour therapy, photodynamic treatment	[40,80]
Multi- stimuli- responsive	Chitosan-HA hybrid with pH/redox sensitivity; alginate-collagen hybrid	Chemotherapeutics, siRNA, and immune adjuvants	Combined responses (e.g., acidic pH + GSH) enable sequential or synergistic release	Precision oncology; combination chemo-immunotherapy	[65,76]

**Table 5:** Selected clinical trials of stimuli-sensitive natural-polymer hydrogel systems.

NCT ID	Hydrogel Type (Natural Polymer)	Indication (Application)	Phase	Status (ClinicalTrials.gov)
**NCT** **05186935**	Hy2Care injectable hydrogel (alginate/gel-like medical hydrogel)	Small knee cartilage defects—injectable cartilage repair	Pivotal/multicentre (investigation)	Active/prospective (investigation listed as pivotal)
**NCT** **00004487**	Chondrocyte alginate gel suspension (alginate)	Autologous chondrocyte implantation for cartilage repair	Phase III (older trial)	Completed (historic pivotal study)
**NCT** **04840147**	JointRep^®^ (thermogel, chitosan-based thermogel)	Repair of chondral lesions (intra-articular application)	Interventional comparative	Recruiting/active (per record)
**NCT** **01226563**	IK-5001 (alginate hydrogel device)	Prevention of ventricular remodeling after myocardial infarction (cardiac repair)	Device trial (early clinical)	Completed/earlier human feasibility
**NCT** **03321396**	Alginate-based injectable (submucosal lift hydrogel)	Assist endoscopic submucosal dissection/mucosal lift (GI endoscopy)	Interventional	Completed/active (trial record)
**NCT** **06028763**	Heparin-conjugated gel (hydrogel scaffold)	Ankle joint cartilage lesions (cartilage repair)	Interventional (developmental)	Recruiting/active (per record)
**NCT** **03686904**	Novel wound gel (hydrogel dressing; composition proprietary)	Chronic wound therapy—infected/chronic wounds	Interventional (device/therapy)	Completed/results reported on safety/tolerability
**NCT** **03190655**	Aluminaid vs. hydrogel wound dressing (hydrogel dressing comparator)	Wound dressing efficacy (various wound types)	Interventional, randomized	Completed/active (results posted)
**NCT** **06309446**	Wound care hydrogel (topical hydrogel)	Assessment of wound healing and local tolerability (abrasive wound model)	Phase I/clinical evaluation	Completed/results reported (tolerability)
**NCT** **01278784**	Chitosan-based eye drops/formulations (chitosan derivative)	Dry eye syndrome/ophthalmic application	Phase 1 (eye drops)	Completed (early human safety)
**NCT** **06394076**	Injectable cosmetic hydrogel (various natural polymer components, e.g., HA blends)	Correction of infraorbital hollowing (aesthetic injectable gel)	Interventional (cosmetic)	Recruiting/active (per record)
**NCT** **03193216**	Adjuvant liquid alginate (alginate liquid)	Oral/GI application (acid reflux—alginate raft formulations)	Interventional (device/therapy)	Completed/registered
**NCT** **06426199**	Chitosan-hyaluronate gel mixture vs. HA	Intra-articular injection/joint applications (comparative)	Interventional (comparative)	Registered/status on record
**NCT** **02877069**	VYC-12 (HA injectable gel)	Cosmetic dermatology (skin filling/fine lines)—injectable HA gel	Interventional/device	Completed (safety/effectiveness)

**Table 6:** Hydrogel biosensor prototypes and their human-testing/clinical status.

Device/Prototype	Polymer Matrix (Primary)	Target Analyte/Sensing Function	Study Type	Clinical Stage/Status	Source (Trial/Paper)
**Smart contact lens (glucose sensor prototypes)**	HA/silicone- hydrogel composites	Tear glucose; intraocular pressure (separate prototypes)	Human correlation/wearable validation studies	Early human studies/pilot validation (human wearability and correlation studies)	[122,123,136]
**Wearable sweat hydrogel sensor** (research prototypes)	Agarose/polysaccharide	Electrolytes, metabolites (e.g., Na^+^, K^+^, lactate)	Human on-body testing/feasibility	Pilot human tests and device validation studies (wearable trials)	[121,137]
**Implantable hydrogel-coated microelectrode sensor (perfusion/EEG coupling)**	Gelatin/collagen or synthetic-natural hybrids	Electrophysiological coupling, tissue mapping	Small clinical feasibility studies/intraoperative use	Early clinical feasibility/pilot use	[120,138,139].
**Transdermal/patch hydrogel biosensor** (biomarker arrays)	HA/cellulose derivatives/alginate hybrid hydrogels	Multiplexed biomarker sensing (e.g., glucose + lactate)/Bacterial presence/protease activity	Human pilot feasibility/wearable validation	Pilot human testing/device validation ongoing	[140,141]
**Hydrogel-based wound sensor strips/dressings**	Alginate/chitosan hybrid dressings	Wound pH, protease activity, bacterial metabolites	Pilot clinical studies/device trials	Early clinical/pilot trials reported (safety, feasibility)	[142,143,144]
**Commercial/clinical hydrogels used for sensing adjuncts (device evaluations)**	Alginate and HA products	Matrices to concentrate analytes for downstream sensors	Clinical device evaluation	Registry/device trials where hydrogel is part of sensing workflow	[59,145,146]
**Emerging smart wound dressing with integrated sensor/readout**	Cellulose and Chitosan/HA hybrid	ROS/pH/protease (combined)	GLP large-animal → planned Phase I	Advanced preclinical; pre-IND/planning first-in-human	[147,148,149,150]

**Study types:** Pilot = small human cohort/feasibility; Clinical feasibility = small exploratory clinical use; Advanced preclinical/GLP = large-animal or GLP toxicology prior to human trials.

**Table 7:** Representative biomedical applications of stimuli-sensitive natural hydrogels.

Application Area	Representative Natural Polymers	Dominant Stimulus Type	Representative Mechanism/Behavior	Biomedical Advantages	Refs.
**Controlled and Targeted Drug Delivery**	Chitosan, Alginate, HA	pH, Redox, Multi-stimuli	Swelling/deswelling or cleavage of crosslinks in response to tumour or endosomal pH	Site-specific drug release, reduced systemic toxicity	[65,66,67,68,69,70,71,72,73,74,75,76]
**Tissue Engineering and Regeneration**	Collagen, Gelatin, Chitosan, Alginate	Enzyme, Temperature, Ionic strength	Enzyme-triggered degradation; thermoresponsive sol–gel transition	ECM mimicry, dynamic stiffness matching	[77,78,79,80,81,82,83,84,85,86,87,88,89,90,91,92,93,94,95,96,97,98]
**Wound Healing and Skin Repair**	Chitosan, Alginate, HA, Cellulose	pH, Redox, Enzymatic	pH-triggered antimicrobial release; ROS-responsive antioxidant delivery	Moist healing, infection control, bioadhesion	[99,100,101,102,103,104,105,106,107,108,109,110,111,112,113,114,115]
**Biosensing and Diagnostics**	HA, Dextran, Cellulose	Biomolecular, pH	Binding-induced swelling/collapse, optical/electrochemical response	Non-invasive sensing, real-time readouts	[113,114,115,116,117,118,119,120,121,122,123,124,125,126,127,128,129,130,131,132,133,134,135,136,137,138,139,140]
**Injectable/In Situ Forming Systems**	Chitosan, Gelatin, HA	Temperature, Enzyme	Thermally triggered sol–gel transition, enzymatic gelation	Minimally invasive delivery, on-site adaptability	[150,151,152,153,154,155,156,157,158,159,160,161,162]

**Table 8:** Mapping Key Challenges to Emerging Solutions.

Challenge	Problem	Clinical Impact	Emerging Trend (Solution)
**Mechanical and Functional Limitations**	Low resistance, or long-term stability under physiological loading.	Limits load-bearing applications (cartilage, tendon), reduces implant durability, increases failure rates.	Hybrid and Nanocomposite Hydrogels (Section 4.1)
**Limited Biostability or Uncontrolled Degradation**	Degradation profiles hard to predict; premature degradation	Reduced therapeutic effect; inconsistent drug release profiles; safety concerns.	Self-Healing and Dynamic Covalent Networks (Section 4.2)
**Suboptimal Stimulus Sensitivity or Specificity**	Response slow, nonspecifically, or irreversibly to stimuli.	Impaired drug release precision; low sensing accuracy; off-target activation.	Multi-Stimuli and Logic-Gated Hydrogels (Section 4.1, Section 4.2 and Section 4.3)
**Complex Biological Interactions and Immunogenicity**	Inflammatory responses depending on source and processing.	Implant rejection; fibrosis; impaired tissue integration.	Bioinspired, Decellularized, or ECM-Mimetic Hydrogels (Section 4.2)
**Regulatory Uncertainty and Lack of Standards**	Unclear classification for regulatory approval.	Delayed translation; higher cost of development; inconsistent quality controls.	Standardized Biomanufacturing and Digital Twins (Section 4.2 and Section 4.4)
**Ethical, Ecological and Supply Chain Constraints**	Environmental impact, sustainability.	Ethical scrutiny; limited scalability; regulatory barriers.	Sustainable Biofabrication and Microbial/Biosynthetic Polymers (Section 4.2)
**Personalization for Patient-Specific Therapy**	Hydrogels cannot be readily individualized.	Limits precision medicine; lower clinical performance.	Personalized and Precision Hydrogel Platforms (Section 4.4)
**Batch-to-Batch Variability and Reproducibility**	Variation depending on source, extraction, and purification.	Unpredictable gelation behavior; regulatory rejection; lack of scalability.	AI-Driven Design and Informatics-Guided Optimization (Section 4.5)

## Data Availability

No new data were created or analyzed in this study. Data sharing is not applicable to this article.

## Abbreviations

The following abbreviations are used in this manuscript:

AI	Artificial intelligence
CM	Computational modeling
DGMH	Gallic acid modified hydrogel microspheres
ECM	Extracellular matrix
EMG	Electromyography
GLP	Good laboratory practice
GMP	Good manufacturing practices
GP	Glycerophosphate
GSH	Glutathione
HA	Hyaluronic acid
IDE	Investigational Device Exemption
IND	Investigational new drug
LCST	Lower critical solution temperature
LSPR	Localized surface plasmon resonance
LV	Left ventricle
ML	Machine learning
MMP	Matrix metalloproteinase
MRI	Magnetic resonance imaging
NCT	National Clinical Trial
PA	Perforating arteries
PPG	Photoplethysmography
QC	Quality control
RGD	Arginylglycylaspartic acid
RNA	Ribonucleic acid
ROS	Reactive oxygen species
TEAi	Transcatheter endocardial alginate hydrogel implantation
UCST	Upper critical solution temperature

## References

[1] Ho T.-C., Chang C.-C., Chan H.-P., Chung T.-W., Shu C.-W., Chuang K.-P., Duh T.-H., Yang M.-H., Tyan Y.-C. (2022). Hydrogels: Properties and Applications in Biomedicine. Molecules.

[2] Thang N.H., Chien T.B., Cuong D.X. (2023). Polymer-Based Hydrogels Applied in Drug Delivery: An Overview. Gels.

[3] Aswathy S.H., Narendrakumar U., Manjubala I. (2020). Commercial hydrogels for biomedical applications. Heliyon.

[4] Chelu M., Calderon Moreno J.M., Musuc A.M., Popa M. (2024). Natural Regenerative Hydrogels for Wound Healing. Gels.

[5] Garcia-Garcia A., Muñana-González S., Lanceros-Mendez S., Ruiz-Rubio L., Alvarez L.P., Vilas-Vilela J.L. (2024). Biodegradable Natural Hydrogels for Tissue Engineering, Controlled Release, and Soil Remediation. Polymers.

[6] Psarrou M., Mitraki A., Vamvakaki M., Kokotidou C. (2023). Stimuli-Responsive Polysaccharide Hydrogels and Their Composites for Wound Healing Applications. Polymers.

[7] Krishani M., Shin W.Y., Suhaimi H., Sambudi N.S. (2023). Development of Scaffolds from Bio-Based Natural Materials for Tissue Regeneration Applications: A Review. Gels.

[8] Neumann M., di Marco G., Iudin D., Viola M., van Nostrum C.F., van Ravensteijn B.G.P., Vermonden T. (2023). Stimuli-responsive hydrogels: The dynamic smart materials of tomorrow. Macromolecules.

[9] El-Husseiny H.M., Mady E.A., Hamabe L., Abugomaa A., Shimada K., Yoshida T., Tanaka T., Yokoi A., Elbadawy M., Tanaka R. (2022). Smart/stimuli-responsive hydrogels: Cutting-edge platforms for tissue engineering and other biomedical applications. Mater. Today Bio.

[10] Bordbar-Khiabani A., Gasik M. (2022). Smart Hydrogels for Advanced Drug Delivery Systems. Int. J. Mol. Sci..

[11] Zhao L., Zhou Y., Zhang J., Liang H., Chen X., Tan H. (2023). Natural Polymer-Based Hydrogels: From Polymer to Biomedical Applications. Pharmaceutics.

[12] Bashir S., Hina M., Iqbal J., Rajpar A.H., Mujtaba M.A., Alghamdi N.A., Wageh S., Ramesh K., Ramesh S. (2020). Fundamental Concepts of Hydrogels: Synthesis, Properties, and Their Applications. Polymers.

[13] Parvin N., Joo S.W., Mandal T.K. (2025). Injectable Biopolymer-Based Hydrogels: A Next-Generation Platform for Minimally Invasive Therapeutics. Gels.

[14] Patra P., Upadhyay T.K., Alshammari N., Saeed M., Kesari K.K. (2024). Alginate-Chitosan Biodegradable and Biocompatible Based Hydrogel for Breast Cancer Immunotherapy and Diagnosis: A Comprehensive Review. ACS Appl. Bio Mater..

[15] Fan R., Cheng Y., Wang R., Zhang T., Zhang H., Li J., Song S., Zheng A. (2022). Thermosensitive Hydrogels and Advances in Their Application in Disease Therapy. Polymers.

[16] Liu M., Zeng X., Ma C., Yi H., Ali Z., Mou X., Li S., Deng Y., He N. (2017). Injectable hydrogels for cartilage and bone tissue engineering. Bone Res..

[17] Nezadi M., Keshvari H., Shokrolahi F., Shokrollahi P. (2024). Injectable, self-healing hydrogels based on gelatin, quaternized chitosan, and laponite as localized celecoxib delivery system for nucleus pulpous repair. Int. J. Biol. Macromol..

[18] Li R., Peng F., Cai J., Yang D., Zhang P. (2020). Redox dual-stimuli responsive drug delivery systems for improving tumor-targeting ability and reducing adverse side effects. Asian J. Pharm. Sci..

[19] Sobczak M. (2022). Enzyme-Responsive Hydrogels as Potential Drug Delivery Systems—State of Knowledge and Future Prospects. Int. J. Mol. Sci..

[20] Lu P., Ruan D., Huang M., Tian M., Zhu K., Gan Z., Zhiao Z. (2024). Harnessing the potential of hydrogels for advanced therapeutic applications: Current achievements and future directions. Signal Transduct. Target. Ther..

[21] Revete A., Aparicio A., Cisterna B.A., Revete J., Luis L., Ibarra E., Segura González E.A., Molino J., Reginensi D. (2022). Advancements in the Use of Hydrogels for Regenerative Medicine: Properties and Biomedical Applications. Int. J. Biomater..

[22] Webber M.J., Pashuck E.T. (2021). (Macro) molecular self-assembly for hydrogel drug delivery. Adv. Drug Deliv. Rev..

[23] Correa S., Grosskopf A.K., Lopez Hernandez H., Chan D., Yu A.C., Stapleton L.M., Appel E.A. (2021). Translational Applications of Hydrogels. Chem. Rev..

[24] Karvinen J., Kellomäki M. (2024). 3D-bioprinting of self-healing hydrogels. Eur. Polym. J..

[25] Fareed M.M., Shityakov S. (2025). Next-Generation Hydrogel Design: Computational Advances in Synthesis, Characterization, and Biomedical Applications. Polymers.

[26] Liu J., Li S., Li S., Tian J., Li H., Pan Z., Lu L., Mao Y. (2024). Recent Advances in Natural-Polymer-Based Hydrogels for Body Movement and Biomedical Monitoring. Biosensors.

[27] Kruczkowska W., Gałęziewska J., Grabowska K., Liese G., Buczek P., Kłosiński K.K., Kciuk M., Pasieka Z., Kołat Ż., Kołat D. (2024). Biomedical Trends in Stimuli-Responsive Hydrogels with Emphasis on Chitosan-Based Formulations. Gels.

[28] Garshasbi H., Salehi S., Naghib S.M., Ghorbanzadeh S., Zhang W. (2023). Stimuli-responsive injectable chitosan-based hydrogels for controlled drug delivery systems. Front. Bioeng. Biotechnol..

[29] Lee K.Y., Mooney D.J. (2012). Alginate: Properties and biomedical applications. Prog. Polym. Sci..

[30] Abka-khajouei R., Tounsi L., Shahabi N., Patel A.K., Abdelkafi S., Michaud P. (2022). Structures, Properties and Applications of Alginates. Mar. Drugs.

[31] Nanda D., Behera D., Pattnaik S.S., Behera A.K. (2025). Advances in natural polymer-based hydrogels: Synthesis, applications, and future directions in biomedical and environmental fields. Discov. Polym..

[32] Malektaj H., Drozdov A.D., de Claville Christiansen J. (2023). Mechanical Properties of Alginate Hydrogels Cross-Linked with Multivalent Cations. Polymers.

[33] Xing H., Lee H., Luo L., Kyriakides T.R. (2020). Extracellular matrix-derived biomaterials in engineering cell function. Biotechnol. Adv..

[34] Zhou H., Li W., Pan L., Zhu T., Zhou T., Xiao E., Wei Q. (2024). Human extracellular matrix (ECM)-like collagen and its bioactivity. Regen. Biomater..

[35] Zöller K., To D., Bernkop-Schnürch A. (2025). Biomedical applications of functional hydrogels: Innovative developments, relevant clinical trials and advanced products. Biomaterials.

[36] Wosicka-Frąckowiak H., Poniedziałek K., Woźny S., Kuprianowicz M., Nyga M., Jadach B., Milanowski B. (2024). Collagen and Its Derivatives Serving Biomedical Purposes: A Review. Polymers.

[37] Xu X., Jha A.K., Harrington D.A., Farach-Carson M.C., Jia X. (2012). Hyaluronic Acid-Based Hydrogels: From a Natural Polysaccharide to Complex Networks. Soft Matter.

[38] Asadi K., Samiraninezhad N., Akbarizadeh A.R., Amini A., Gholami A. (2024). Stimuli-responsive hydrogel based on natural polymers for breast cancer. Front. Chem..

[39] Xu Q., Torres J.E., Hakim M., Babiak P.M., Pal P., Battistoni C.M., Nguyen M., Panitch A., Solorio L., Liu J.C. (2021). Collagen- and hyaluronic acid-based hydrogels and their biomedical applications. Mater. Sci. Eng. R Rep..

[40] Protsak I.S., Morozov Y.M. (2025). Fundamentals and Advances in Stimuli-Responsive Hydrogels and Their Applications: A Review. Gels.

[41] Veloso S.R.S., Azevedo A.G., Teixeira P.F., Fernandes C.B.P. (2023). Cellulose Nanocrystal (CNC) Gels: A Review. Gels.

[42] Yao Q., Lan Q.H., Jiang X., Du C.C., Zhai Y.Y., Shen X., Xu H.L., Xiao J., Kou L., Zhao Y.Z. (2020). Bioinspired biliverdin/silk fibroin hydrogel for antiglioma photothermal therapy and wound healing. Theranostics.

[43] Lyu Y., Liu Y., He H., Wang H. (2023). Application of Silk-Fibroin-Based Hydrogels in Tissue Engineering. Gels.

[44] Madappura A.P., Madduri S. (2023). A comprehensive review of silk-fibroin hydrogels for cell and drug delivery applications in tissue engineering and regenerative medicine. Comput. Struct. Biotechnol. J..

[45] Mohamed M.A., Fallahi A., El-Sokkary A.M.A., Salehi S., Akl M.A., Jafari A., Tamayol A., Fenniri H., Khademhosseini A., Andreadis S.T. (2019). Stimuli-responsive hydrogels for manipulation of cell microenvironment: From chemistry to biofabrication technology. Prog. Polym. Sci..

[46] Gao K., Xu K. (2025). Advancements and Prospects of pH-Responsive Hydrogels in Biomedicine. Gels.

[47] Ng H.W., Zhang Y., Naffa R., Prabakar S. (2020). Monitoring the Degradation of Collagen Hydrogels by Collagenase Clostridium histolyticum. Gels.

[48] Patterson J., Siew R., Herring S.W., Lin A.S., Guldberg R., Stayton P.S. (2010). Hyaluronic acid hydrogels with controlled degradation properties for oriented bone regeneration. Biomaterials.

[49] Kilic Boz R., Aydin D., Kocak S., Golba B., Sanyal R., Sanyal A. (2022). Redox-Responsive Hydrogels for Tunable and “On-Demand” Release of Biomacromolecules. Bioconjugate Chem..

[50] Abed H.F., Abuwatfa W.H., Husseini G.A. (2022). Redox-Responsive Drug Delivery Systems: A Chemical Perspective. Nanomaterials.

[51] Rana M.M., De la Hoz Siegler H. (2024). Evolution of Hybrid Hydrogels: Next-Generation Biomaterials for Drug Delivery and Tissue Engineering. Gels.

[52] Bustamante-Torres M., Romero-Fierro D., Arcentales-Vera B., Palomino K., Magaña H., Bucio E. (2021). Hydrogels Classification According to the Physical or Chemical Interactions and as Stimuli-Sensitive Materials. Gels.

[53] Ghorbani M., Vasheghani-Farahani E., Azarpira N., Hashemi-Najafabadi S., Ghasemi A. (2023). Dual-crosslinked in-situ forming alginate/silk fibroin hydrogel with potential for bone tissue engineering. Biomater. Adv..

[54] Mushtaq F., Raza Z.A., Batool S.R., Zahid M., Onder O.C., Rafique A., Nazeer M.A. (2022). Preparation, properties, and applications of gelatin-based hydrogels (GHs) in the environmental, technological, and biomedical sectors. Int. J. Biol. Macromol..

[55] Johari N., Moroni L., Samadikuchaksaraei A. (2020). Tuning the conformation and mechanical properties of silk fibroin hydrogels. Eur. Polym. J..

[56] Degirmenci A., Sanyal R., Sanyal A. (2024). Metal-Free Click-Chemistry: A Powerful Tool for Fabricating Hydrogels for Biomedical Applications. Bioconjugate Chem..

[57] Mahmoudi C., Tahraoui Douma N., Mahmoudi H., Iurciuc C.E., Popa M. (2024). Hydrogels Based on Proteins Cross-Linked with Carbonyl Derivatives of Polysaccharides, with Biomedical Applications. Int. J. Mol. Sci..

[58] Tong Z., Jin L., Oliveira J.M., Reis R.L., Zhong Q., Mao Z., Gao C. (2021). Adaptable hydrogel with reversible linkages for regenerative medicine: Dynamic mechanical microenvironment for cells. Bioact. Mater..

[59] Clegg J.R., Adebowale K., Zhao Z., Mitragotri S. (2024). Hydrogels in the clinic: An update. Bioeng. Transl. Med..

[60] Peppas N., Hilt J., Khademhosseini A., Langer R. (2006). Hydrogels in Biology and Medicine: From Molecular Principles to Bionanotechnology. Adv. Mater..

[61] Coviello T., Matricardi P., Marianecci C., Alhaique F. (2007). Polysaccharide hydrogels for modified release formulations. J. Control. Release.

[62] Shoulders M.D., Raines R.T. (2009). Collagen structure and stability. Annu. Rev. Biochem..

[63] Hoare T.R., Kohane D.S. (2008). Hydrogels in drug delivery: Progress and challenges. Polymer.

[64] Hoffman A.S. (2002). Hydrogels for biomedical applications. Adv. Drug Deliv. Rev..

[65] Pashkina E., Bykova M., Berishvili M., Lazarev Y., Kozlov V. (2025). Hyaluronic Acid-Based Drug Delivery Systems for Cancer Therapy. Cells.

[66] Delgado-Pujol E.J., Martínez G., Casado-Jurado D., Vázquez J., León-Barberena J., Rodríguez-Lucena D., Torres Y., Alcudia A., Begines B. (2025). Hydrogels and Nanogels: Pioneering the Future of Advanced Drug Delivery Systems. Pharmaceutics.

[67] Zhu T., Mao J., Cheng Y., Liu H., Lv L., Ge M., Li S., Huang J., Chen Z., Li H. (2019). Recent Progress of Polysaccharide-Based Hydrogel Interfaces for Wound Healing and Tissue Engineering. Adv. Mater. Interfaces.

[68] Li X., Xu X., Xu M., Geng Z., Ji P., Liu Y. (2023). Hydrogel systems for targeted cancer therapy. Front. Bioeng. Biotechnol..

[69] Firouzabadi B.M., Gigliobianco M.R., Agas D., Sabbieti M.G., Alimenti C., Devi L.S., Casadidio C., Martino P.D., Censi R. (2025). Stimuli-sensitive hyaluronic acid hydrogels for localized and controlled release of antibodies. Eur. J. Pharm. Biopharm..

[70] Tian H., Yang L. (2025). Chitosan-Based Smart Hydrogel Platform for Targeted Intravesical Delivery in Bladder Cancer Therapy. J. Polym. Sci..

[71] Kobrin R.L., Mantooth S.M., Mulry A.L., Zaharoff D.J., Zaharoff D.A. (2025). Chitosan–Glycerol Injectable Hydrogel for Intratumoral Delivery of Macromolecules. Gels.

[72] Hariyadi D.M., Islam N. (2020). Current Status of Alginate in Drug Delivery. Adv. Pharmacol. Pharm. Sci..

[73] Abasalizadeh F., Moghaddam S.V., Alizadeh E., Akbari E., Kashani E., Fazljou S.M.B., Torbati M., Akbarzadeh A. (2020). Alginate-based hydrogels as drug delivery vehicles in cancer treatment and their applications in wound dressing and 3D bioprinting. J. Biol. Eng..

[74] Guo A., Cao Q., Fang H., Tian H. (2025). Recent advances and challenges of injectable hydrogels in drug delivery. J. Control. Release.

[75] Román-Guerrero A., Cortés-Camargo S., Alpizar-Reyes E., Fabela-Morón M.F., Cruz-Olivares J., Velázquez-Gutiérrez S.K., Pérez-Alonso C. (2025). Chemically Modified Alginate-Based Hydrogel-Matrices in Drug Delivery. Macromol.

[76] Luo W., Cheng W., Ni S., Zhu X., Wu M. (2025). A review of stimuli-responsive hydrogels for RNA delivery: From material innovations to clinical barriers. Int. J. Biol. Macromol..

[77] Kharaziha M., Baidya A., Annabi N. (2021). Rational Design of Immunomodulatory Hydrogels for Chronic Wound Healing. Adv. Mater..

[78] Ni X., Xing X., Deng Y., Li Z. (2023). Applications of Stimuli-Responsive Hydrogels in Bone and Cartilage Regeneration. Pharmaceutics.

[79] Yamashita Y., Hosoya K., Fujiwara Y., Saito Y., Yoshida M., Matsune S., Okubo K., Takei T. (2025). Injectable Chitosan Hydrogel Particles as Nasal Packing Materials After Endoscopic Sinus Surgery for Treatment of Chronic Sinusitis. Gels.

[80] Liu C., Liu C., Liu Z., Shi Z., Liu S., Wang X., Wang X., Huang F. (2023). Injectable thermogelling bioadhesive chitosan-based hydrogels for efficient hemostasis. Int. J. Biol. Macromol..

[81] Gomez-Florit M., Pardo A., Domingues R.M.A., Graça A.L., Babo P.S., Reis R.L., Gomes M.E. (2020). Natural-Based Hydrogels for Tissue Engineering Applications. Molecules.

[82] Alsaikhan F., Farhood B. (2024). Recent advances on chitosan/hyaluronic acid-based stimuli-responsive hydrogels and composites for cancer treatment: A comprehensive review. Int. J. Biol. Macromol..

[83] Atwal A., Dale T.P., Snow M., Forsyth N.R., Davoodi P. (2023). Injectable hydrogels: An emerging therapeutic strategy for cartilage regeneration. Adv. Colloid Interface Sci..

[84] Zhao W., Jin X., Cong Y., Liu Y., Fu J. (2013). Degradable natural polymer hydrogels for articular cartilage tissue engineering. J. Chem. Technol. Biotechnol..

[85] Kang Y., Guan Y., Li S. (2024). Innovative hydrogel solutions for articular cartilage regeneration: A comprehensive review. Int. J. Surg..

[86] van de Looij S.M., de Jong O.G., Vermonden T., Lorenowicz M.J. (2023). Injectable hydrogels for sustained delivery of extracellular vesicles in cartilage regeneration. J. Control. Release.

[87] Advanced Cartilage Treatment with Injectable-Hydrogel Validation of the Effect (ACTIVE). NCT05186935. NCT05186935.

[88] Xu Q., Xiao Z., Yang Q., Yu T., Deng X., Chen N., Huang Y., Wang L., Guo J., Wang J. (2024). Hydrogel-based cardiac repair and regeneration function in the treatment of myocardial infarction. Mater. Today Bio.

[89] Wang B., Lee R.J., Tao L. (2023). First-in-human transcatheter endocardial alginate-hydrogel implantation for the treatment of heart failure. Eur. Heart J..

[90] Zhang L., Shao L., Li J., Zhang Y., Shen Z. (2024). Annexin A1-Loaded Alginate Hydrogel Promotes Cardiac Repair via Modulation of Macrophage Phenotypes after Myocardial Infarction. ACS Biomater. Sci. Eng..

[91] Bai X., Gao M., Syed S., Zhuang J., Xu X., Zhang X.-Q. (2018). Bioactive hydrogels for bone regeneration. Bioact. Mater..

[92] Xue C., Chen L., Wang N., Chen H., Xu W., Xi Z., Sun Q., Kang R., Xie L., Liu X. (2024). Stimuli-responsive hydrogels for bone tissue engineering. Biomater. Transl..

[93] Wang Z., Zhai B., Sun J., Zhang X., Zou J., Shi Y., Guo D. (2024). Recent advances of injectable in situ-forming hydrogels for preventing postoperative tumor recurrence. Drug Deliv..

[94] Schwab A., Levato R., D’Este M., Piluso S., Eglin D., Malda J. (2020). Printability and Shape Fidelity of Bioinks in 3D Bioprinting. Chem. Rev..

[95] Unagolla J.M., Jayasuriya A.C. (2020). Hydrogel-based 3D bioprinting: A comprehensive review on cell-laden hydrogels, bioink formulations, and future perspectives. Appl. Mater. Today.

[96] Liu F., Chen Q., Liu C., Ao Q., Tian X., Fan J., Tong H., Wang X. (2018). Natural Polymers for Organ 3D Bioprinting. Polymers.

[97] Briones Y., Pascua B., Tiangco N., Crisostomo I., Casiguran S., Remenyi R. (2025). Assessing the landscape of clinical and observational trials involving bioprinting: A scoping review. 3D Print. Med..

[98] Distler T., McDonald K., Heid S., Karakaya E., Detsch R., Boccaccini A.R. (2020). Ionically and Enzymatically Dual Cross-Linked Oxidized Alginate Gelatin Hydrogels with Tunable Stiffness and Degradation Behavior for Tissue Engineering. ACS Biomater. Sci. Eng..

[99] Guo W., Ding X., Zhang H., Liu Z., Han Y., Wei Q., Okoro O.V., Shavandi A., Nie L. (2024). Recent Advances of Chitosan-Based Hydrogels for Skin-Wound Dressings. Gels.

[100] Zhang X., Liang Y., Huang S., Guo B. (2024). Chitosan-based self-healing hydrogel dressing for accelerated wound healing. Adv. Colloid Interface Sci..

[101] Ren M., Yao J., Yang D., Zhu J., Dai K., Zhong Y., Zhu J., Tang L., Xu Y., Yu J. (2025). Chitosan hydrogels loaded with Cu_3_SnS_4_ NSs for the treatment of second-degree burn wounds. Sci. Rep..

[102] Gou Y., Hu L., Liao X., He J., Liu F. (2024). Advances of antimicrobial dressings loaded with antimicrobial agents in infected wounds. Front. Bioeng. Biotechnol..

[103] Rajinikanth B.S., Rajkumar D.K.K. (2024). Chitosan-Based Biomaterial in Wound Healing: A Review. Cureus.

[104] Gao Z., Golland B., Tronci G., Thornton P.D. (2019). A redox-responsive hyaluronic acid-based hydrogel for chronic wound management. J. Mater. Chem. B.

[105] Cherri M., Stergiou P.S., Ahmadian Z., Povolotsky T.L., Thongrom B., Fan X., Mohammadifar E., Haag R. (2024). Redox-Responsive Hydrogels Loaded with an Antibacterial Peptide as Controlled Drug Delivery for Healing Infectious Wounds. Adv. Healthc. Mater..

[106] Wu L., Zhou Y., Zhang Y., Hu J., Ikegami Y., Aishima S., Ijima H. (2025). Fast Wound Healing with a New Functional Hyaluronic Acid Dual Network Hydrogel. Gels.

[107] Zhao H., Wu Y., Xie Y., Li Y., Chen C., Li C., Yang F., Zhang D., Wang Y., Yuan J. (2024). Hydrogel dressings for diabetic foot ulcer: A systematic review and meta-analysis. Diabetes Obes. Metab..

[108] Mazurek L., Kuś M., Jurak J., Rybka M., Kuczeriszka M., Stradczuk-Mazurek M., Konop M. (2025). Biomedical potential of alginate wound dressings—From preclinical studies to clinical applications: A review. Int. J. Biol. Macromol..

[109] Jia S., Wang J., Wang X., Liu X., Li S., Li Y., Li J., Wang J., Man S., Guo Z. (2023). Genetically encoded in situ gelation redox-responsive collagen-like protein hydrogel for accelerating diabetic wound healing. Biomater. Sci..

[110] Zhu A., Chen B., Ma J., Wang J., Tang R., Liu L., Sun W., Zheng X., Pan G. (2025). Application of Antimicrobial Peptides in Wound Dressings. Drug Des. Dev. Ther..

[111] Zheng W., Wang L., Jiao H., Wu Z., Zhao Q., Lin T., Ma H., Zhang Z., Xu X., Cao J. (2023). A cost-effective, fast cooling, and efficient anti-inflammatory multilayered topological hydrogel patch for burn wound first aid. Chem. Eng. J..

[112] Wang J., Ye J., Li Z., Li X., Luo Y., Zhou Z., Liu C., Xu T., Zhang X. (2025). An Integrated Janus Bioelectronic Bandage for Unidirectional Pumping and Monitoring of Wound Exudate. Nano Lett..

[113] Zhang H., Wu C. (2023). 3D printing of biomaterials for vascularized and innervated tissue regeneration. Int. J. Bioprint..

[114] Ganesan O., Orgill D.P. (2024). An Overview of Recent Clinical Trials for Diabetic Foot Ulcer Therapies. J. Clin. Med..

[115] Mallanagoudra P., M Ramakrishna S.S., Jaiswal S., Keshava Prasanna D., Seetharaman R., Palaniappan A., Kini S. (2025). Progressive Hydrogel Applications in Diabetic Foot Ulcer Management: Phase-Dependent Healing Strategies. Polymers.

[116] Quazi M.Z., Hwang J., Song Y., Park N. (2023). Hydrogel-Based Biosensors for Effective Therapeutics. Gels.

[117] Herrmann A., Haag R., Schedler U. (2021). Hydrogels and Their Role in Biosensing Applications. Adv. Healthc. Mater..

[118] Sun S., Chen J. (2024). Recent Advances in Hydrogel-Based Biosensors for Cancer Detection. ACS Appl. Mater. Interfaces.

[119] Wu J., Hong J., Gao X., Wang Y., Wang W., Zhang H., Park J., Shi W., Guo W. (2025). Recent Progress in Flexible Wearable Sensors Utilizing Conductive Hydrogels for Sports Applications: Characteristics, Mechanisms, and Modification Strategies. Gels.

[120] Yang G., Qiu Y., Pang B., Guo W., Liu S., Zheng Q., Zhou S., Tian J., Liu Y., Wu H. (2025). A reusable hydrogel biosensor array with electrically responsive hydrogel interfaces for noninvasive locating of perforating arteries. Sci. Adv..

[121] Nie N.-Y., Chen Y.-C. (2023). Bioadhesive hydrogel flexible laser for sweat sensing based on liquid crystal microdroplets. arXiv.

[122] Park W., Seo H., Kim J., Hong Y.-M., Song H., Joo B.J., Kim S., Yae C.-G., Kim J., Jin J. (2024). In-depth correlation analysis between tear glucose and blood glucose using a wireless smart contact lens. Nat. Commun..

[123] Keum D.H., Kim S.K., Koo J., Lee G.H., Jeon C., Mok J.W., Mun B.H., Lee K.J., Kamrani E., Joo C.K. (2020). Wireless smart contact lens for diabetic diagnosis and therapy. Sci. Adv..

[124] Sun X., Ding C., Qin M., Li J. (2024). Hydrogel-Based Biosensors for Bacterial Infections. Small.

[125] Qi M., Ke Y., Li Y., Li P., Zhou H., Zhang X., Chen J., Meng J. (2025). Au@Pt nanozyme-based smart hydrogel for visual detection of hyaluronic acid. Sens. Actuators B Chem..

[126] Calderón Moreno J.M., Chelu M., Popa M. (2025). Eco-Friendly Conductive Hydrogels: Towards Green Wearable Electronics. Gels.

[127] Yu J., Wan R., Tian F., Cao J., Wang W., Liu Q., Yang H., Liu J., Liu X., Lin T. (2024). 3D Printing of Robust High-Performance Conducting Polymer Hydrogel-Based Electrical Bioadhesive Interface for Soft Bioelectronics. Small.

[128] Hao S., Dai R., Fu Q., Wang Y., Zhang X., Li H., Liu X., Yang J. (2023). A Robust and Adhesive Hydrogel Enables Interfacial Coupling for Continuous Temperature Monitoring. Adv. Funct. Mater..

[129] Wan R., Yu J., Quan Z., Ma H., Li J., Tian F., Wang W., Sun Y., Liu J., Gao D. (2024). A reusable, healable, and biocompatible PEDOT:PSS hydrogel-based electrical bioadhesive interface for high-resolution electromyography monitoring and time–frequency analysis. Chem. Eng. J..

[130] Hong S., Yu T., Wang Z., Lee C.H. (2025). Biomaterials for reliable wearable health monitoring: Applications in skin and eye integration. Biomaterials.

[131] Qiu J., Ma H., Yao M., Song M., Zhang L., Xu J., Liu X., Lu B. (2024). Design of a supersoft, ultra-stretchable, and 3D printable hydrogel electrical bioadhesive interface for electromyography monitoring. Supramol. Mater..

[132] Shi S., Wang Y., Ye Z., Xie H., Liu C., Liao J., Zhao D., Sun Q., Shamshina J.L., Shen X. (2025). Dual-Working-Pattern Nanosheet-Based Hydrogel Sensors for Constructing Human-Machine and Physiological-Electric Interfaces. Adv. Sci..

[133] Elsherif M., Moreddu R., Alam F., Salih A.E., Ahmed I., Butt H. (2022). Wearable Smart Contact Lenses for Continual Glucose Monitoring: A Review. Front. Med..

[134] Sutar P., Pethe A., Kumar P., Tripathi D., Maity D. (2025). Hydrogel Innovations in Biosensing: A New Frontier for Pancreatitis Diagnostics. Bioengineering.

[135] Omidian H., Chowdhury S.D., Akhzarmehr A. (2024). Hydrogels in biosensing and medical diagnostics. J. Bioact. Compat. Polym..

[136] Clinical Performance of 59% Hioxifilcon A vs. Marketed Hydrogel Contact Lenses. NCT04986644. NCT04986644.

[137] Wang J., Luo Y., Zhou Z., Xiao J., Xu T., Zhang X. (2024). Epidermal wearable optical sensors for sweat monitoring. Commun. Mater..

[138] Cheng S., Zhu R., Xu X. (2024). Hydrogels for next generation neural interfaces. Commun. Mater..

[139] Spencer K.C., Sy J.C., Santoro M., Mahmood M., Cohen R.E., Cima M.J. (2017). Characterization of mechanically matched hydrogel coatings for neural probes. Sci. Rep..

[140] Dai Y., Nolan J., Madsen E., Fratus M., Lee J., Zhang J., Lim J., Hong S., Alam M.A., Linnes J.C. (2023). Wearable sensor patch with hydrogel microneedles for in situ analysis of interstitial fluid. ACS Appl. Mater. Interfaces.

[141] Chu X., Wang H., Liu Z., Tang J. (2024). Magnetic porous hydrogel-enhanced wearable patch sensor for sweat zinc-ion monitoring. Sensors.

[142] Zhang Y., Li T., Zhao C., Li J., Huang R., Zhang Q., Li Y., Li X. (2021). An integrated smart sensor dressing for real-time wound microenvironment monitoring and promoting angiogenesis and wound healing. Front. Cell Dev. Biol..

[143] Jin S., Newton M.A.A., Cheng H., Zhang Q., Gao W., Zheng Y., Lu Z., Dai Z., Zhu J. (2023). Progress of Hydrogel Dressings with Wound Monitoring and Treatment Functions. Gels.

[144] Cui Y., Niu Q., Hu Z., He H., Wei X., Wang M., Chen X.-L., Wang X. (2025). Multifunctional nanozyme-hydrogel system in bacterial-infected wounds. Cell Biomater..

[145] Roden R.K., Raines K.S., Hollingsworth J.W., Stadelman K.M., Swan A.J., Miller J.J. (2024). Human tear-film protein sampling using soft contact lenses. Clin. Proteom..

[146] PMS Study of Silver I Alginate Non-Woven Dressing (Hydro-Alginate). NCT05690685. NCT05690685.

[147] Pang Q., Yang F., Jiang Z., Wu K., Hou R., Zhu Y. (2023). Smart wound dressing for advanced wound management: Real-time monitoring and on-demand treatment. Mater. Des..

[148] Zuo W., Wei K., Zhang X., Wang D., Gong H., Zhang Y., Wang H. (2024). A Multifunctional Nanozyme Hydrogel with Antibacterial, Antioxidative, and Photo-Induced Nitric Oxide-Supplying Properties for Promoting Infected Wound Healing. Pharmaceutics.

[149] Eskilson O., Wiman E., Reustle N., Langwagen J., Sotra Z., Svärd A., Selegård R., Baş Y., Berglund L., Oksman K. (2025). Nanocellulose Wound Dressings with Integrated Protease Sensors for Detection of Wound Pathogens. ACS Sens..

[150] Pang Q., Lou D., Li S., Wang G., Qiao B., Dong S., Ma L., Gao C., Wu Z. (2020). Smart Flexible Electronics-Integrated Wound Dressing for Real-Time Monitoring and On-Demand Treatment of Infected Wounds. Adv. Sci..

[151] Rahmanian-Devin P., Baradaran Rahimi V., Askari V.R. (2021). Thermosensitive Chitosan-β-Glycerophosphate Hydrogels as Targeted Drug Delivery Systems: An Overview on Preparation and Their Applications. Adv. Pharmacol. Pharm. Sci..

[152] Bhuiyan M.H., Clarkson A.N., Ali M.A. (2023). Optimization of thermoresponsive chitosan/β-glycerophosphate hydrogels for injectable neural tissue engineering application. Colloids Surf. B Biointerfaces.

[153] Zhu Y., Matsumura Y., Wagner W.R. (2017). Ventricular wall biomaterial injection therapy after myocardial infarction: Advances in material design, mechanistic insight and early clinical experiences. Biomaterials.

[154] Yan Y., Chen Y., Huang L., Cai M., Yin X., Zhu Y.Z., Ye L. (2025). Bioengineered In Situ-Forming Hydrogels as Smart Drug Delivery Systems for Postoperative Breast Cancer Immunotherapy: From Material Innovation to Clinical Translation. J. Funct. Biomater..

[155] Fan S., Liu Q., Dong J., Ai X., Li J., Huang W., Sun T. (2024). In situ forming an injectable hyaluronic acid hydrogel for drug delivery and synergistic tumor therapy. Heliyon.

[156] Frey N., Linke A., Süselbeck T., Müller-Ehmsen J., Vermeersch P., Schoors D., Rosenberg M., Bea F., Tuvia S., Leor J. (2014). Intracoronary delivery of injectable bioabsorbable scaffold (IK-5001) to treat left ventricular remodeling after ST-elevation myocardial infarction: A first-in-man study. Circ. Cardiovasc. Interv..

[157] Cattelan G., Guerrero Gerbolés A., Foresti R., Pramstaller P.P., Rossini A., Miragoli M., Caffarra Malvezzi C. (2020). Alginate Formulations: Current Developments in the Race for Hydrogel-Based Cardiac Regeneration. Front. Bioeng. Biotechnol..

[158] IK-5001 for the Prevention of Remodeling of the Ventricle and Congestive Heart Failure After Acute Myocardial Infarction (PRESERVATION-1). NCT01226563. NCT01226563.

[159] Wu J., Chen Q., Deng C., Xu B., Zhang Z., Yang Y., Lu T. (2020). Exquisite design of injectable Hydrogels in Cartilage Repair. Theranostics.

[160] Yao P., Tan Z., Weng B., Wang X., Wang H., Yang G., Sun F., Zhao Y. (2024). Locally Injectable Chitosan/β-Glycerophosphate Hydrogel Doped with Triptolide–Human Serum Albumin Nanoparticles for Treating Rheumatoid Arthritis. Pharmaceuticals.

[161] Chang Y.-F., Cheng Y.-H., Ko Y.-C., Chiou S.-H., Liu C.J.-L. (2022). Development of topical chitosan/ β-glycerophosphate-based hydrogel loaded with levofloxacin in the treatment of keratitis: An ex-vivo study. Heliyon.

[162] Li G., Feng J., He F., Xu G., Wu C., Ma X., Qiao Y., Luo Z., Du P. (2025). HA-PAs hydrogel for enhanced cartilage repair in early osteoarthritis: A novel minimally invasive treatment strategy. Biomed. Pharmacother..

[163] Yin B., Gosecka M., Bodaghi M., Crespy D., Youssef G., Dodda J.M., Wong S.H.D., Imran A.B., Gosecki M., Jobdeedamrong A. (2024). Engineering multifunctional dynamic hydrogel for biomedical and tissue regenerative applications. Chem. Eng. J..

[164] Alshangiti D.M., El-Damhougy T.K., Zaher A., Madani M., Mohamady Ghobashy M. (2023). Revolutionizing biomedicine: Advancements, applications, and prospects of nanocomposite macromolecular carbohydrate-based hydrogel biomaterials: A review. RSC Adv..

[165] Guo X., Liu H., Nail A., Meng D., Zhu L., Li C., Li H. (2025). Design and Application of Stimuli-Responsive Nanocomposite Hydrogels: A Review. Macromol. Rapid Commun..

[166] Gaharwar A.K., Peppas N.A., Khademhosseini A. (2014). Nanocomposite hydrogels for biomedical applications. Biotechnol. Bioeng..

[167] Chandra D.K., Kumar A., Mahapatra C. (2025). Smart nano-hybrid metal-organic frameworks: Revolutionizing advancements, applications, and challenges in biomedical therapeutics and diagnostics. Hybrid Adv..

[168] Cai W., Wang J., Chu C., Chen W., Wu C., Liu G. (2018). Metal-Organic Framework-Based Stimuli-Responsive Systems for Drug Delivery. Adv. Sci..

[169] Cui X., Ruan Q., Zhuo X., Xia X., Hu J., Fu R., Li Y., Wang J., Xu H. (2023). Photothermal Nanomaterials: A Powerful Light-to-Heat Converter. Chem. Rev..

[170] Li J., Zeng H., Zeng Z., Zeng Y., Xie T. (2021). Promising Graphene-Based Nanomaterials and Their Biomedical Applications and Potential Risks: A Comprehensive Review. ACS Biomater. Sci. Eng..

[171] Ghandforoushan P., Alehosseini M., Golafshan N., Castilho M., Dolatshahi-Pirouz A., Hanaee J., Davaran S., Orive G. (2023). Injectable hydrogels for cartilage and bone tissue regeneration: A review. Int. J. Biol. Macromol..

[172] Sung B., Shaffer S., Sittek M., Alboslemy T., Kim C., Kim M.H. (2016). Alternating Magnetic Field-Responsive Hybrid Gelatin Microgels for Controlled Drug Release. J. Vis. Exp..

[173] Agrawal A., Hussain C.M. (2025). State-of-the-art in functionalized 3D/4D-printed magnetic hydrogels for environmental and biomedical applications. Adv. Colloid Interf. Sci..

[174] Domingo-Diez J., Foti A., Casanova-Carvajal Ó., Marrodán L., Granado N., Satriano C., Martínez-Murillo R., Serrano-Olmedo J.J., Ramos-Gómez M. (2025). Effect of Photothermal Therapy Using Gold Nanoparticles Conjugated with Hyaluronic Acid in an Intracranial Murine Glioblastoma Model. Int. J. Nanomed..

[175] Umar A.K., Limpikirati P.K., Rivai B., Ardiansah I., Sriwidodo S., Luckanagul J.A. (2025). Complexed hyaluronic acid-based nanoparticles in cancer therapy and diagnosis: Research trends by natural language processing. Heliyon.

[176] Raia N.R., Partlow B.P., McGill M., Kimmerling E.P., Ghezzi C.E., Kaplan D.L. (2017). Enzymatically crosslinked silk-hyaluronic acid hydrogels. Biomaterials.

[177] Quan L., Xin Y., Wu X., Ao Q. (2022). Mechanism of Self-Healing Hydrogels and Application in Tissue Engineering. Polymers.

[178] Talebian S., Mehrali M., Taebnia N., Pennisi C.P., Kadumudi F.B., Foroughi J., Hasany M., Nikkhah M., Akbari M., Orive G. (2019). Self-Healing Hydrogels: The Next Paradigm Shift in Tissue Engineering?. Adv. Sci..

[179] Cho S., Hwang S.Y., Oh D.X., Park J. (2021). Recent progress in self-healing polymers and hydrogels based on reversible dynamic B–O bonds: Boronic/boronate esters, borax and benzoraborole. J. Mater. Chem. A.

[180] Xu J., Hsu S.-H. (2023). Self-healing hydrogel as an injectable implant: Translation in brain diseases. J. Biomed. Sci..

[181] Xu Y., Li Y., Chen Q., Fu L., Tao L., Wei Y. (2018). Injectable and Self-Healing Chitosan Hydrogel Based on Imine Bonds: Design and Therapeutic Applications. Int. J. Mol. Sci..

[182] Hong C., Chung H., Lee G., Kim D., Jiang Z., Kim S.H., Lee K. (2024). Remendable Cross-Linked Alginate/Gelatin Hydrogels Incorporating Nanofibers for Wound Repair and Regeneration. Biomacromolecules.

[183] Gil E.S., Hudson S.M. (2007). Effect of silk fibroin interpenetrating networks on swelling/deswelling kinetics and rheological properties of poly(N-isopropylacrylamide) hydrogels. Biomacromolecules.

[184] Zhang H., Xu D., Zhang Y., Li M., Chai R. (2022). Silk fibroin hydrogels for biomedical applications. Smart Med..

[185] Mukherjee S., Krishnan A., Athira R.K., Kasoju N., Sah M.K., Sah M.K., Kasoju N., Mano J.F. (2022). Silk fibroin and silk sericin in skin tissue engineering and wound healing: Retrospect and prospects. Natural Polymers in Wound Healing and Repair.

[186] Patil P.P., Reagan M.R., Bohara R.A. (2020). Silk fibroin and silk-based biomaterial derivatives for ideal wound dressings. Int. J. Biol. Macromol..

[187] Yu G., Niu C., Liu J., Wu J., Jin Z., Wang Y., Zhao K. (2023). Preparation and Properties of Self-Cross-Linking Hydrogels Based on Chitosan Derivatives and Oxidized Sodium Alginate. ACS Omega.

[188] Tian X., Wen Y., Zhang Z., Zhu J., Song X., Phan T.T., Li J. (2025). Recent advances in smart hydrogels derived from polysaccharides and their applications for wound dressing and healing. Biomaterials.

[189] Han S., Gao L., Dou X., Wang Z., Yang K., Li D., Yuan Y., Xing C., Jiang B., Tian Y. (2024). Chiral Hydrogel Nerve Conduit Boosts Peripheral Nerve Regeneration via Regulation of Schwann Cell Reprogramming. ACS Nano.

[190] Wang M., Deng Z., Guo Y., Xu P. (2022). Designing functional hyaluronic acid-based hydrogels for cartilage tissue engineering. Mater. Today Bio.

[191] An C., Li H., Zhao Y., Zhang S., Zhao Y., Zhang Y., Yang J., Zhang L., Ren C., Zhang Y. (2023). Hyaluronic acid-based multifunctional carriers for applications in regenerative medicine: A review. Int. J. Biol. Macromol..

[192] Zhang M., Ye Q., Zhu Z., Shi S., Xu C., Xie R., Li Y. (2024). Hyaluronic Acid-Based Dynamic Hydrogels for Cartilage Repair and Regeneration. Gels.

[193] Di Marzio N., Eglin D., Serra T., Moroni L. (2020). Bio-Fabrication: Convergence of 3D Bioprinting and Nano-Biomaterials in Tissue Engineering and Regenerative Medicine. Front. Bioeng. Biotechnol..

[194] Cui R., Li S., Li T., Gou X., Jing T., Zhang G., Wei G., Jin Z., Xiong X., Qu S. (2023). Natural polymer-derived hydrogel bioink with enhanced thixotropy improves printability and cellular preservation in 3D bioprinting. J. Mater. Chem. B.

[195] Zhang H., Zhou Z., Zhang F., Wan C. (2024). Hydrogel-Based 3D Bioprinting Technology for Articular Cartilage Regenerative Engineering. Gels.

[196] Hasany M., Talebian S., Sadat S., Ranjbar N., Mehrali M., Wallace G.G., Mehrali M. (2021). Synthesis, properties, and biomedical applications of alginate methacrylate (ALMA)-based hydrogels: Current advances and challenges. Appl. Mater. Today.

[197] Uysal B., Madduma-Bandarage U.S.K., Jayasinghe H.G., Madihally S. (2025). 3D-Printed Hydrogels from Natural Polymers for Biomedical Applications: Conventional Fabrication Methods, Current Developments, Advantages, and Challenges. Gels.

[198] Yang Z., Yi P., Liu Z., Zhang W., Mei L., Feng C., Tu C., Li Z. (2022). Stem Cell-Laden Hydrogel-Based 3D Bioprinting for Bone and Cartilage Tissue Engineering. Front. Bioeng. Biotechnol..

[199] Guo A., Zhang S., Yang R., Sui C. (2024). Enhancing the mechanical strength of 3D printed GelMA for soft tissue engineering applications. Mater. Today Bio.

[200] Kotani T., Mubarok W., Hananouchi T., Sakai S. (2023). Horseradish Peroxidase-Mediated Bioprinting via Bioink Gelation by Alternately Extruded Support Material. ACS Biomater. Sci. Eng..

[201] Honkamäki L., Kulta O., Puistola P., Hopia K., Emeh P., Isosaari L., Mörö A., Narkilahti S. (2024). Hyaluronic Acid-Based 3D Bioprinted Hydrogel Structure for Directed Axonal Guidance and Modeling Innervation In Vitro. Adv. Healthc. Mater..

[202] Nie X., Tang Y., Wu T., Zhao X., Xu Z., Yang R., Sun Y., Wu B., Han Q., Hui J. (2024). 3D printing sequentially strengthening high-strength natural polymer hydrogel bilayer scaffold for cornea regeneration. Regen. Biomater..

[203] Hull S.M., Brunel L.G., Heilshorn S.C. (2022). 3D Bioprinting of Cell-Laden Hydrogels for Improved Biological Functionality. Adv. Mater..

[204] Martins C.F., García-Astrain C., Conde J., Liz-Marzán L.M. (2024). Nanocomposite hydrogel microneedles: A theranostic toolbox for personalized medicine. Drug Deliv. Transl. Res..

[205] Nelson B.E., Naing A., Fu S., Sheth R.A., Murthy R., Piha-Paul S. (2024). Potentiating intratumoral therapy with immune checkpoint inhibitors: Shifting the paradigm of multimodality therapeutics. Immunooncol. Technol..

[206] Gu Z., Fu J., Lin H., He Y. (2020). Development of 3D bioprinting: From printing methods to biomedical applications. Asian J. Pharm. Sci..

[207] Regeneration of Knee and Ankle Cartilage from Autologous Cartilage Mini-Grafts (from the Patient’s Own Cells). NCT06897098. NCT06897098.

[208] Yang Y., Zeng W., Huang P., Zeng X., Mei L. (2021). Smart Materials for Drug Delivery and Cancer Therapy. View.

[209] Guo Y., Chen Y., Wu Y., Zhu Y., Luo S., Shen J., Luo Y. (2024). Injectable pH-responsive polypeptide hydrogels for local delivery of doxorubicin. Nanoscale Adv..

[210] Gierlich P., Donohoe C., Behan K., Kelly D.J., Senge M.O., Gomes-da-Silva L.C. (2024). Antitumor Immunity Mediated by Photodynamic Therapy Using Injectable Chitosan Hydrogels for Intratumoral and Sustained Drug Delivery. Biomacromolecules.

[211] Ali A., Saroj S., Saha S., Gupta S.K., Rakshit T., Pal S. (2023). Glucose-Responsive Chitosan Nanoparticle/Poly(vinyl alcohol) Hydrogels for Sustained Insulin Release In Vivo. ACS Appl. Mater. Interfaces.

[212] Pal S., Rakshit T., Saha S., Jinagal D. (2025). Glucose-Responsive Materials for Smart Insulin Delivery: From Protein-Based to Protein-Free Design. ACS Mater. Au.

[213] Banach Ł., Williams G.T., Fossey J.S. (2021). Insulin Delivery Using Dynamic Covalent Boronic Acid/Ester-Controlled Release. Adv. Therap..

[214] Laycock B.G., Chan C.M., Halley P.J. (2024). A review of computational approaches used in the modelling, design, and manufacturing of biodegradable and biobased polymers. Prog. Polym. Sci..

[215] Quesada-Pérez M., Ramos J., Forcada J., Martín-Molina A. (2012). Computer simulations of thermo-sensitive microgels: Quantitative comparison with experimental swelling data. J. Chem. Phys..

[216] Li S., Zhang H., Chen K., Jin M., Vu S.H., Jung S., He N., Zheng Z., Lee M.S. (2022). Application of chitosan/alginate nanoparticle in oral drug delivery systems: Prospects and challenges. Drug Deliv..

[217] Bisotti F., Pizzetti F., Storti G., Rossi F. (2022). Mathematical modelling of cross-linked polyacrylic-based hydrogels: Physical properties and drug delivery. Drug Deliv. Transl. Res..

[218] Chopra H., Annu, Shin D.K., Munjal K., Priyanka, Dhama K., Emran T.B. (2023). Revolutionizing clinical trials: The role of AI in accelerating medical breakthroughs. Int. J. Surg..

[219] Luo T., Tan B., Zhu L., Wang Y., Liao J. (2022). A review on the design of hydrogels with different stiffness and their effects on tissue repair. Front. Bioeng. Biotechnol..

[220] Xu F., Dawson C., Lamb M., Mueller E., Stefanek E., Akbari M., Hoare T. (2022). Hydrogels for Tissue Engineering: Addressing Key Design Needs Toward Clinical Translation. Front. Bioeng. Biotechnol..

[221] Guan X., Avci-Adali M., Alarçin E., Cheng H., Kashaf S.S., Li Y., Chawla A., Jang H.L., Khademhosseini A. (2017). Development of hydrogels for regenerative engineering. Biotechnol. J..

[222] Pires P.C., Mascarenhas-Melo F., Pedrosa K., Lopes D., Lopes J., Macário-Soares A., Peixoto D., Giram P.S., Veiga F., Paiva-Santos A.C. (2023). Polymer-based biomaterials for pharmaceutical and biomedical applications: A focus on topical drug administration. Eur. Polym. J..

[223] Pohan G., Mattiassi S., Yao Y., Zaw A.M., Anderson D.E.J., Cutiongco M.F.A., Hinds M.T., Yim E.K.F. (2020). Effect of Ethylene Oxide Sterilization on Polyvinyl Alcohol Hydrogel Compared with Gamma Radiation. Tissue Eng. Part A.

[224] Haridas N., Rosemary M.J. (2019). Effect of steam sterilization and biocompatibility studies of hyaluronic acid hydrogel for viscosupplementation. Polym. Degrad. Stab..

[225] (2016). Medical Devices—Quality Management Systems—Requirements for Regulatory Purposes.

[226] Li Z., Song P., Li G., Han Y., Ren X., Bai L., Su J. (2024). AI-energized hydrogel design, optimization and application in biomedicine. Mater. Today Bio.

[227] U.S. Food & Drug Administration Combination Products—Guidance & Regulatory Information. FDA. 2024–2025. https://www.fda.gov/combination-products/guidance-regulatory-information.

[228] Reis M.E., Bettencourt A., Ribeiro H.M. (2022). The regulatory challenges of innovative customised combination products. Front. Med..

[229] Ou B.S., Saouaf O.M., Baillet J., Appel E.A. (2022). Sustained delivery approaches to improving adaptive immune responses. Adv. Drug Deliv. Rev..

[230] Schuh J.C.L., Funk K.A. (2019). Compilation of International Standards and Regulatory Guidance Documents for Evaluation of Biomaterials, Medical Devices, and 3-D Printed and Regenerative Medicine Products. Toxicol. Pathol..

[231] (2025). Biological Evaluation of Medical Devices Part 1: Requirements and General Principles for the Evaluation of Biological Safety Within a Risk Management Process.

[232] Duasa J., Husin A.M., Thaker A.M.T.M., Rahman M.P. (2022). An alternative source of collagen for Muslim consumers: Halal and environmental concerns. J. Islam. Mark..

[233] Romano S., Yazdanpanah S., Petillo O., Conte R., Sepe F., Peluso G., Calarco A. (2025). Sustainable Hydrogels for Medical Applications: Biotechnological Innovations Supporting One Health. Gels.

[234] (2024). EU-Wide End-of-Waste Criteria for Plastic Waste.

[235] Liu J., Du C., Huang W., Lei Y. (2024). Injectable smart stimuli-responsive hydrogels: Pioneering advancements in biomedical applications. Biomater. Sci..

[236] Gonçalves M., Figueira P., Maciel D., Rodrigues J., Qu X., Liu C., Tomás H., Li Y. (2014). pH-sensitive Laponite^®^/doxorubicin/alginate nanohybrids with improved anticancer efficacy. Acta Biomater..

[237] Lin X., Ma Q., Su J., Wang C., Kankala R.K., Zeng M., Lin H., Zhou S.F. (2019). Dual-Responsive Alginate Hydrogels for Controlled Release of Therapeutics. Molecules.

[238] Çetin D.P., Seçme M., İlhan H., Sağlam N. (2025). Alginate and chitosan-coated ferulic acid-loaded selenium nanoparticles: Synthesis, characterization, and anticancer activity against MDA-MB-231 breast cancer cells. Med. Oncol..

[239] Lee S.J., Nah H., Heo D.N., Kim K.-H., Seok J.M., Heo M., Moon H.-J., Lee D., Lee J.S., An S.Y. (2020). Induction of osteogenic differentiation in a rat calvarial bone defect model using an n situ forming graphene oxide incorporated glycol chitosan/oxidized hyaluronic acid injectable hydrogel. Carbon.

[240] Omer S.A., McKnight K.H., Young L.I., Song S. (2023). Stimulation strategies for electrical and magnetic modulation of cells and tissues. Cell Regen..

[241] Londhe P.V., Londhe M.V., Salunkhe A.B., Laha S.S., Mefford O.T., Thorat N.D., Khot V.M. (2025). Magnetic hydrogel (MagGel): An evolutionary pedestal for anticancer therapy. Coord. Chem. Rev..

[242] Cui X., Li J., Hartanto Y., Durham M., Tang J., Zhang H., Hooper G., Lim K., Woodfield T. (2020). Advances in Extrusion 3D Bioprinting: A Focus on Multicomponent Hydrogel-Based Bioinks. Adv. Healthc. Mater..

[243] Zennifer A., Manivannan S., Sethuraman S., Kumbar S.G., Sundaramurthi D. (2022). 3D bioprinting and photocrosslinking: Emerging strategies & future perspectives. Biomater. Adv..

[244] Tripathi S., Mandal S.S., Bauri S., Maiti P. (2022). 3D bioprinting and its innovative approach for biomedical applications. MedComm.

[245] Ma X., Sekhar K.P.C., Zhang P., Cui J. (2024). Advances in stimuli-responsive injectable hydrogels for biomedical applications. Biomater. Sci..

[246] Perin F., Ouyang L., Lim K.S., Motta A., Manigli D., Moroni L., Mota C. (2025). Bioprinted Constructs in the Regulatory Landscape: Current State and Future Perspectives. Adv. Mater..

[247] Lu J., Gao Y., Cao C., Wang H., Ruan Y., Qin K., Liu H., Wang Y., Yang P., Liu Y. (2025). 3D bioprinted scaffolds for osteochondral regeneration: Advancements and applications. Mater. Today Bio.

[248] Zhou H., Zhu Y., Yang B., Huo Y., Yin Y., Jiang X., Ji W. (2024). Stimuli-responsive peptide hydrogels for biomedical applications. J. Mater. Chem. B.

[249] Liu X., Hu L., Chen S., Ran Y., Pang J., Wu S. (2025). Machine learning analysis for the rheological mechanism of polysaccharide colloids. J. Mol. Liq..

[250] Xu S., Chen X., Wang S., Chen Z., Pan P., Huang Q. (2024). Integrating machine learning for the optimization of polyacrylamide/alginate hydrogel. Regen. Biomater..

[251] Mtshali S., Jacobs B.A. (2025). Machine learning-based prediction of pharmacokinetic parameters for individualized drug dosage optimization. Int. J. Inf. Tecnol..

[252] Xin H., Maruf D.S.A.A., Akin-Ige F., Amin S. (2025). Stimuli-responsive hydrogels for skin wound healing and regeneration. Emergent Mater..

[253] Castiello C., Junghanns P., Mergel A., Jacob C., Ducho C., Valente S., Rotili D., Fioravanti R., Zwergel C., Mai A. (2023). GreenMedChem: The challenge in the next decade toward eco-friendly compounds and processes in drug design. Green Chem..

[254] Alonso-Cuevas C.F., Ramírez-Guzmán N., Serna-Cock L., Guancha-Chalapud M., Aguirre-Joya J.A., Aguillón-Gutiérrez D.R., Claudio-Rizo A., Torres-León C. (2025). From Agro-Industrial Waste to Natural Hydrogels: A Sustainable Alternative to Reduce Water Use in Agriculture. Gels.

[255] Chelu M., Popa M., Calderón Moreno J.M. (2025). Applications of Hydrogels in Emergency Therapy. Gels.

[256] Chelu M., Popa M., Calderón Moreno J.M. (2025). Next-Generation Natural Hydrogels in Oral Tissue Engineering. Pharmaceutics.

[257] Tang N., Zheng Y., Jiang X., Zhou C., Jin H., Jin K., Wu W., Haick H. (2021). Wearable Sensors and Systems for Wound Healing-Related pH and Temperature Detection. Micromachines.

